# New Irreversible α‐l‐Iduronidase Inhibitors and Activity‐Based Probes

**DOI:** 10.1002/chem.201804662

**Published:** 2018-11-26

**Authors:** Marta Artola, Chi‐Lin Kuo, Stephen A. McMahon, Verena Oehler, Thomas Hansen, Martijn van der Lienden, Xu He, Hans van den Elst, Bogdan I. Florea, Allison R. Kermode, Gijsbert A. van der Marel, Tracey M. Gloster, Jeroen D. C. Codée, Herman S. Overkleeft, Johannes M. F. G. Aerts

**Affiliations:** ^1^ Department of Bio-organic Synthesis Leiden Institute of Chemistry Leiden University Einsteinweg 55 2333 CC Leiden The Netherlands; ^2^ Department of Medical Biochemistry Leiden Institute of Chemistry Leiden University Einsteinweg 55 2333 CC Leiden The Netherlands; ^3^ Biomedical Sciences Research Complex School of Biology University of St Andrews North Haugh St Andrews Fife KY16 9ST UK; ^4^ Department of Biological Sciences Simon Fraser University 8888 University Drive Burnaby BC V5A 1S6 Canada

**Keywords:** activity-based protein profiling, conformational analysis, cyclophellitol aziridines, glycosidase, irreversible inhibitors

## Abstract

Cyclophellitol aziridines are potent irreversible inhibitors of retaining glycosidases and versatile intermediates in the synthesis of activity‐based glycosidase probes (ABPs). Direct 3‐amino‐2‐(trifluoromethyl)quinazolin‐4(3*H*)‐one‐mediated aziridination of l‐*ido*‐configured cyclohexene has enabled the synthesis of new covalent inhibitors and ABPs of α‐l‐iduronidase, deficiency of which underlies the lysosomal storage disorder mucopolysaccharidosis type I (MPS I). The iduronidase ABPs react covalently and irreversibly in an activity‐based manner with human recombinant α‐l‐iduronidase (rIDUA, Aldurazyme^®^). The structures of IDUA when complexed with the inhibitors in a non‐covalent transition state mimicking form and a covalent enzyme‐bound form provide insights into its conformational itinerary. Inhibitors **1**–**3** adopt a half‐chair conformation in solution (^4^H_3_ and ^3^H_4_), as predicted by DFT calculations, which is different from the conformation of the Michaelis complex observed by crystallographic studies. Consequently, **1**–**3** may need to overcome an energy barrier in order to switch from the ^4^H_3_ conformation to the transition state (^2, 5^B) binding conformation before reacting and adopting a covalent ^5^S_1_ conformation. rIDUA can be labeled with fluorescent Cy5 ABP **2**, which allows monitoring of the delivery of therapeutic recombinant enzyme to lysosomes, as is intended in enzyme replacement therapy for the treatment of MPS I patients.

## Introduction

**Figure 1:**
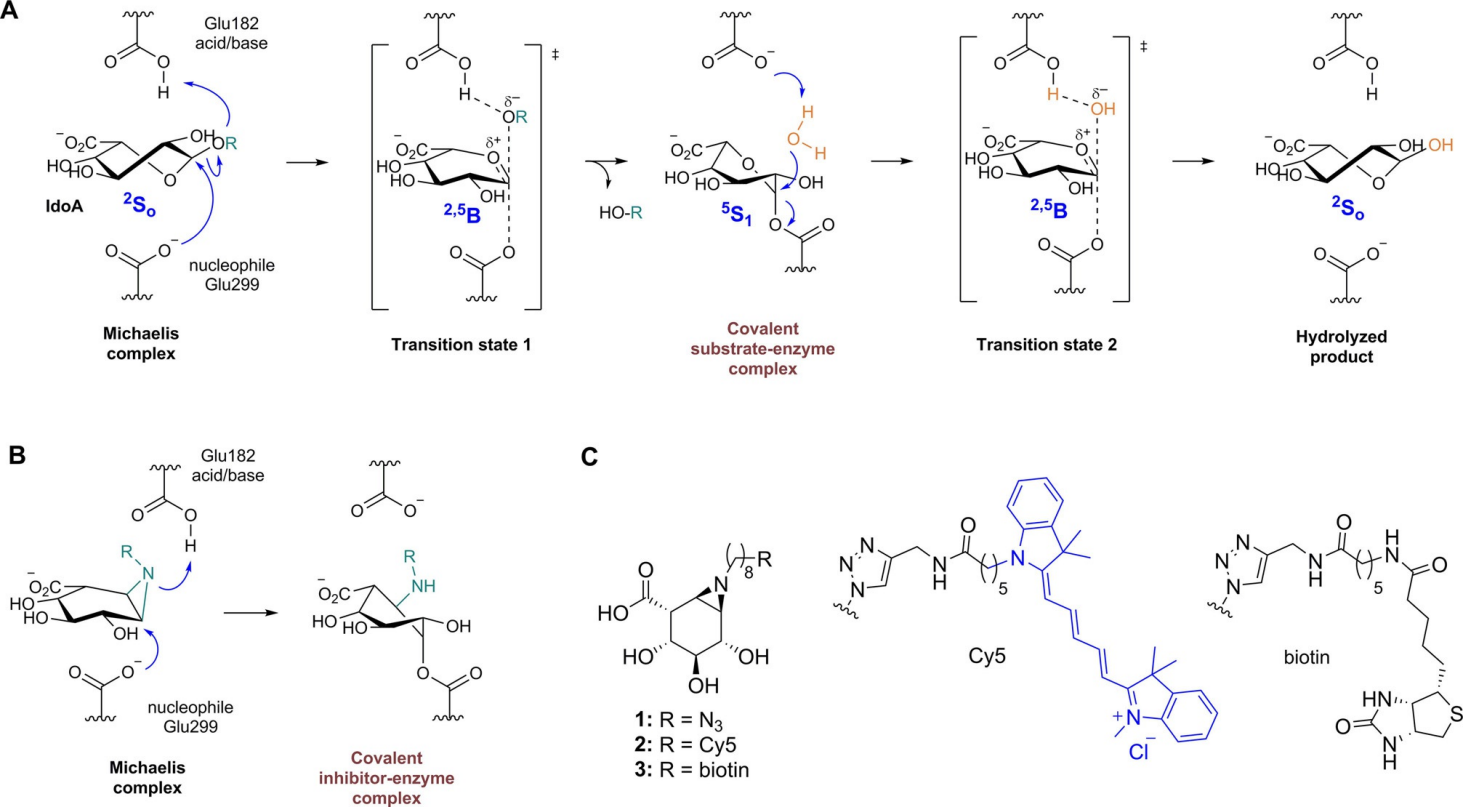
A) Koshland double‐displacement mechanism employed by retaining α‐l‐iduronidase, showing the ^2^S_0_→^2, 5^B→^5^S_1_→^2, 5^B→^2^S_0_ conformational reaction itinerary from the Michaelis complex, transition state 1, covalent substrate–enzyme intermediate, and transition state 2, to the hydrolyzed product. B) Proposed inhibition mechanism of aziridine‐based inhibitor **1** and ABPs **2** and **3**. C) Chemical structures of α‐l‐iduronic‐configured mechanism‐based irreversible inhibitor **1** and ABPs **2** and **3** described in this work.

Human α‐l‐iduronidase (IDUA), which belongs to the GH39 family in the Carbohydrate Active EnZyme (CAZy) classification,^1^, ^2^ hydrolyzes terminal non‐reducing α‐l‐iduronic acid residues in glycoaminoglycans (GAGs), including dermatan sulfate (DS) and heparan sulfate (HS), through a two‐step Koshland double‐displacement mechanism (Figure 1 A).^3^, ^4^, ^5^, ^6^ The active site of the enzyme contains a carboxylic acid/carboxylate pair that acts as an acid/base (Glu188) and a nucleophilic (Glu299) catalyst. Protonation of the exocyclic oxygen by the acid/base residue and concomitant nucleophilic attack at the anomeric carbon by Glu299 leads to S_N_2 displacement of the aglycon, yielding a covalent enzyme–substrate complex with inversion of stereochemistry at the anomeric carbon. In the next step, a water molecule enters the enzyme active site, where it is deprotonated by the acid/base (Glu188) and then hydrolyzes the enzyme–substrate intermediate in a reversal of steps, again with inversion of anomeric configuration. The conformational change of IDUA from Michaelis complex to transition state (TS) and enzyme–inhibitor covalent complex has recently been suggested to follow a ^2^S_o_→^2, 5^B→^5^S_1_ itinerary.^5^, ^7^, ^8^ This catalytic pathway was predicted on the basis of the structures of Michaelis complexes with (2*R*, 3*R*, 4*R*, 5*S*)‐2‐carboxy‐3,4,5‐trihydroxypiperidine (IdoA‐DNJ) and 5‐fluoro‐α‐l‐idopyranosyluronic acid fluoride (5F‐IdoAF) as reversible inhibitors and the 2‐deoxy‐2‐fluoro‐α‐l‐idopyranosyluronic (2F‐IdoA)–enzyme covalent complex intermediate.

Mutations in the gene encoding IDUA may result in mucopolysaccharidosis type I (MPS I), which is a rare autosomal inherited recessive disease that leads to toxic accumulation of HS and DS. MPS I is a devastating disease that affects around 1 in 100,000 individuals and is classified as attenuated MPS I and more severe MPS I (traditionally categorized from less to more severe form as Scheie, Hurler–Scheie, or Hurler disease) to distinguish between disease severity and therapeutic options.^8^ Children with severe MPS I are treated at a young age by hematopoietic cell transplantation (HCT).^9^ Enzyme replacement therapy (ERT) with recombinant human α‐l‐iduronidase (rIDUA, Aldurazyme^®^) is considered as a treatment for non‐neurological manifestations of MPS I.^10^ There is consensus among treating clinicians that the impact of ERT with rIDUA is greatest when the treatment is initiated early in the disease progression. An obvious prerequisite for effectiveness is the successful targeting of infused rIDUA to lysosomes in multiple cell types, and for this purpose a detailed understanding of rIDUA targeting is still urgently needed. Besides MPS I, lysosomal α‐l‐iduronidase is indirectly involved in two other inherited lysosomal storage disorders, mucolipidosis II (ML II) and III α/β. Here, a deficiency in the generation of mannose‐6‐phosphate (M6P) moieties in *N*‐linked glycans of newly formed lysosomal enzymes impairs their correct routing to lysosomes and therefore these, including α‐l‐iduronidase, are largely erroneously secreted.^11^

Herein, we report the synthesis of irreversible IDUA inhibitors and activity‐based probes (ABPs) bearing an α‐l‐iduronic‐configured cyclophellitol aziridine as an electrophilic “warhead”. Functionalization of the aziridine with a Cy5 fluorophore (for gel and/or cell imaging) or biotin (for chemical proteomics studies) afforded valuable tools (Figure 1 B, C) for the study of α‐l‐iduronidase in vitro and in situ, structural analysis, and for monitoring rIDUA uptake and trafficking to lysosomes, as is revealed here.

## Results and Discussion

### Synthesis of α‐l‐iduronic‐configured inhibitors and ABPs

**Figure 2:**
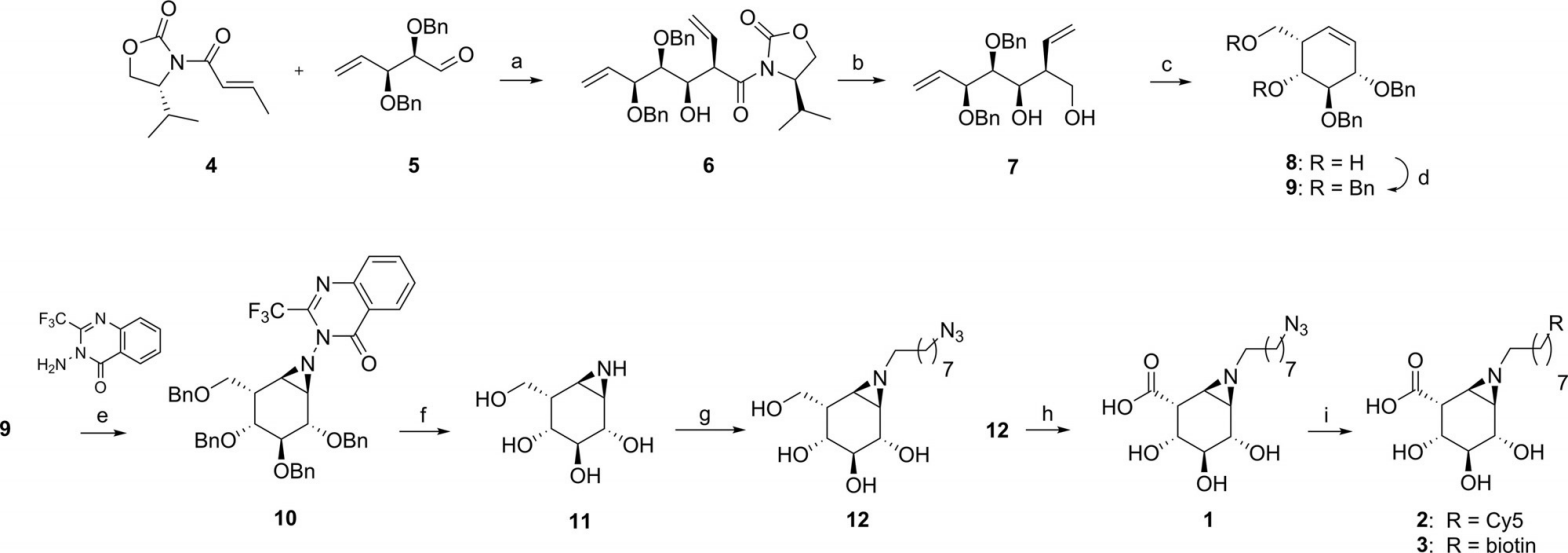
Synthesis of α‐l‐iduronic‐configured inhibitors and ABPs **1**–**3**. Reagents and conditions: a) DBBT, Et_3_N, CH_2_Cl_2_, −78 °C to −20 °C, 5 h, 60 %; b) LiBH_4_, THF, rt, 2 h, 99 %; c) Grubbs II catalyst, CH_2_Cl_2_, 40 °C, 18 h, 98 %; d) BnBr, TBAI, NaH, DMF, rt, 18 h, 79 %; e) PhI(OAc)_2_, CH_2_Cl_2_, rt, 48 h, 43 %; f) Li, NH_3_, THF, −60 °C, 1 h, 93 %; g) 8‐azido‐1‐iodooctane, K_2_CO_3_, DMF, 55 °C, 24 h, **12**: 22 %; h) TEMPO, NaBr, NaOCl, H_2_O, 0 °C, 3 h, 14 %; i) CuSO_4_, NaAsc, rt, 18–48 h, **2**: 22 %, **3**: 34 %.

Synthetic strategies to assemble cyclophellitol derivatives often involve different configurations of functionalized cyclohexenes as starting materials. Based on the synthesis of d‐*galacto*‐ and l‐*fuco*‐configured cyclohexenes described by Llebaria and co‐workers^12^ and our group,^13^ we reasoned that reaction of dibenzylated aldehyde **5** 14 with chiral Evans’ oxazolidinone **4** should provide the l *‐ido*‐configured cyclohexene by *syn*‐aldol addition (Figure 2). Indeed, asymmetric aldol condensation catalyzed by dibutylboryl triflate at low temperatures (−78 °C to −20 °C) proceeded stereoselectively to provide the desired aldol product **6** in 60 % yield. During the reaction, the non‐reactive terminal alkene derived from isomerization of acrylamide **4** was observed as a major side product. Reduction of oxazolidinone **6** with LiBH_4_ followed by Grubbs II‐catalyzed metathesis afforded the desired l‐*ido*‐configured cyclohexene **8** in excellent yield.

We then attempted olefin aziridination of l‐*ido*‐configured cyclohexene **8** with the specific aim of obtaining the α‐stereoisomer, and thus potential inhibitors and probes to study IDUA. Recently, Llebaria and co‐workers reported the first *N*‐aminoaziridine covalent glycosidase inhibitors, which were prepared by stereoselective hydrogen‐bonding‐guided aziridination using 3‐amino‐2‐ethylquinazolin‐4(3*H*)‐one (Et‐Q‐NH_2_).^12^ Because such hydrogen‐bond‐mediated aziridination of cyclohexene **8** would generate the undesired β‐diastereoisomer, the free alcohol groups in **8** were benzylated with benzyl bromide and sodium hydride to generate cyclohexene **9**. When the direct azidirination of **9** was performed with in situ generated CF_3_‐Q‐NHOAc complex, the desired α‐aziridine **10** was obtained in 43 % yield together with 32 % recovered starting material (Figure 2). This result implied that hydrogen bonding is not required for a productive aziridination, provided that the double bond is freely accessible. Removal of the CF_3_‐Q and benzyl groups was achieved in one step by Birch reduction using lithium and liquid ammonia at −78 °C. After quenching the reaction with H_2_O, CF_3_‐Q‐derived impurities precipitated and were filtered off. Aziridine **11** was purged of lithium hydroxide by cation‐exchange chromatography with Amberlite H^+^ resin, and the fully deprotected cyclitol aziridine was obtained in 93 % yield (Figure 2).

The α‐l‐configuration of aziridine **11** was established by ^1^H NMR analysis, and the experimental coupling constants were compared with the corresponding calculated values obtained from DFT calculations (see the Supporting Information).

Aziridine **11** was then alkylated with 8‐azido‐1‐iodooctane and K_2_CO_3_ or acylated with 8‐azidooctanoic acid and EEDQ to afford intermediates **12** or **13**, respectively, which were purified by reversed‐phase column chromatography. Oxidation of C‐6 proved to be challenging due to instability of the aziridine under acidic or basic conditions. Aziridine **1** was obtained in 14 % yield by oxoammonium‐catalyzed oxidation, maintaining the reaction and HPLC‐MS purification at basic pH. Final click reaction with Cy5‐ and biotin‐substituted alkynes afforded the desired ABPs **2** and **3**.

### In vitro inhibition and labeling of recombinant human α‐l‐iduronase with inhibitors 1–3

**Figure 3:**
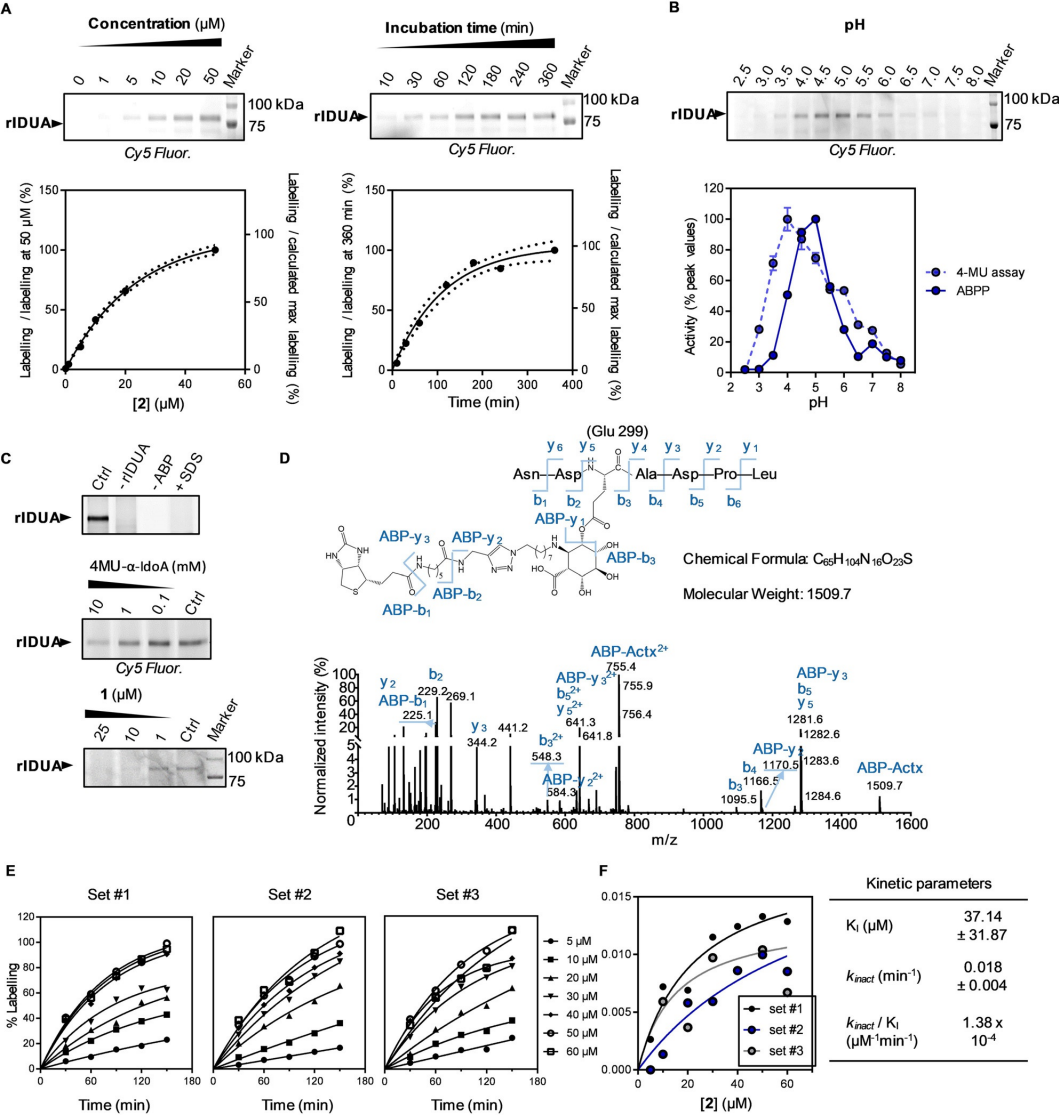
Labeling of rIDUA with ABPs **2** and **3**. A) Cy5 ABP **2** labels rIDUA (10 ng) in a concentration‐dependent (left, at 4 h incubation time) and time‐dependent (right, at 50 μm) manner. Labeling signals were quantified (below each gel image) and fitted with a one‐phase association equation. Dotted lines represent 95 % CI. B) ABP **2** labels rIDUA in a pH‐dependent manner. The quantified labeling signals were compared to data obtained with 4‐MU substrate assay (below; error range=SD from technical triplicates). C) ABP **2** labels rIDUA and negative controls: without rIDUA, without ABP **2** or with SDS (upper panel), competition with 4‐MU‐α‐l‐iduronide (middle panel) or **1** (lower panel). D) MS/MS pattern of a sample containing rIDUA Asn297–Leu303 active site peptide labeled with biotin ABP **3** at Glu299, showing peaks corresponding to the detected fragments. Actx=active site peptide. E) Percentages of rIDUA labeling at different time points and at different concentrations of ABP **2**. Data were quantified from three sets of fluorescent gels containing rIDUA labeled with ABP **2** under the depicted conditions to derive a rate constant *k* for each ABP **2** concentration. F) Left, *k* vs. [inhibitor] plot. Data were curve‐fitted with the Michaelis–Menten equation to obtain kinetic parameters. Right, calculated kinetic parameters for ABP **2** labeling of rIDUA. Error range=SD from the three sets.

We examined the inhibition potencies of compounds **1**–**3** by incubating them for 60 min with human recombinant α‐l‐iduronidase (rIDUA, Genzyme) at pH 4.5 in competition with the fluorogenic substrate 4‐methylumbelliferyl‐α‐iduronide (4‐MU‐IdoA). Compounds **1**–**3** inhibited rIDUA with apparent half‐maximum inhibitory concentrations in the micromolar range (IC_50_ values of **1**: 40.6±17.0 μm, **2**: 58.1±6.66 μm, and **3**: 65.1±5.73 μm). Intermediate **11** showed no activity, in line with the role of the carboxylate group at C5 for IDUA binding in a positively charged enzymatic pocket formed by the Arg363 and Lys264 side chains and the main‐chain NH groups of Gly305 and Trp306.^8^ Surprisingly, alkyl aziridine **12** displayed an apparent IC_50_ of 12.2±3.24 μm, suggesting that the carboxylate group may not be essential for binding. These results are also in line with previous findings that *N*‐alkyl aziridines display improved binding potency towards glycosidases (Figure S1).^15^ rIDUA was labeled by Cy5 ABP **2** in a concentration‐ and time‐dependent manner, consistent with the irreversible inhibition mechanism of these analogues, with optimal labeling at 50 μm and 120 min incubation, as visualized by SDS‐PAGE (Figure 3 A). The optimum pH for labeling with ABP **2** was determined as 4.5–5.0, consistent with the reported optimum pH for enzymatic activity (Figure 3 B).^2^ In addition, competitive activity‐based protein profiling (ABPP) showed competition in a concentration‐dependent manner with 4‐MU‐IdoA as well as with inhibitor **1**, illustrating the applicability of this probe for the screening of new inhibitors (Figure 3 C). We also tested the stability of the covalent enzyme–inhibitor complex and observed that rIDUA remained inactivated for at least 100 h (Figure S2).

We next determined kinetic parameters for rIDUA labeling/inhibition by ABP **2** at various concentrations (5–60 μm) and different incubation times (30–150 min) (Figure 3 E, F; see also Figure S3 for SDS‐PAGE gels). ABP **2** irreversibly inhibited rIDUA, with an initial binding constant (*K*_I_) of 37.1 μm and an inactivation rate constant (*k*_inact_) of 0.018 min^−1^, showing this compound to be a less potent inactivator than other ABPs for related glycosidases, such as β‐glucuronidases^15^ (GUSB and HPSE), β‐glucosidases^16^, ^17^ (GBA 1, 2, and 3), α‐glucosidases^18^ (GAA and GANAB), and α‐galactosidase^19^ (GLA).

### Analysis of the covalent ABP 3–IDUA complex by LC‐MS/MS

To further demonstrate the covalent binding to the catalytically active amino acid, we incubated biotinylated ABP **3** with rIDUA. After chymotryptic digestion and affinity enrichment by streptavidin beads, the resulting ABP **3**‐labeled chymotryptic peptides were analyzed by nanoscale liquid chromatography coupled with tandem mass spectrometry (nano‐LC‐MS/MS). A fragment of the IDUA nucleophile (Glu299) covalently attached to ABP **3** was detected by MS/MS fragmentation of the 7‐amino‐acid peptide containing the nucleophilic residue (Figure 3 D; Figures S5 and S6, and Table S1).

### Activity‐based protein profiling of IDUA in homogenates of fibroblasts

We attempted to visualize endogenous IDUA in lysates of cultured normal human dermal fibroblasts (NHDF) and concentrated human urine, with and without pre‐purification by concanavalin A beads. Unfortunately, no clear labeling was observed with ABP **2**, and many non‐specific bands were detected by SDS‐PAGE that could not be competed by inhibitor **1** nor ABP **3**. We compared the specific glycosidase activity and ABP labeling of β‐glucocerebrosidase (GBA) vs. IDUA with those of the corresponding Cy5 ABPs (ABP **JJB367** 20 vs. **2**) in recombinant enzymes and human fibroblast lysates (NHDF). It was observed that while it is theoretically possible to detect IDUA in fibroblast lysates (calculated amount of endogenous IDUA was 16 fmol vs. 10 fmol of rIDUA detected by ABP **2**), its detection in NHDF lysates is still challenging due to nonspecific labeling of other proteins at the required concentration of ABP **2** (25–50 μm) (Figure S4). Moreover, the calculated amount of IDUA was 1.5× lower than that of GBA in fibroblast lysates, which might also hamper IDUA detection (Figure S4). We conclude that SDS‐PAGE IDUA labeling in cell lysates is not feasible due to a combination of low abundance of IDUA in the tested samples and moderate activities of inhibitors **1**–**3**.

### Conformations of α‐l‐*ido*‐aziridine 11 and α‐l‐*idoA*‐aziridine by DFT calculations

We studied the conformational preferences of α‐l‐*ido*‐aziridine **11** and α‐l‐*idoA* methylated aziridine as simplified representations of inhibitors **1**–**3**. A conformer distribution search in Spartan 14^21^ and further optimization with Gaussian 09^22^ by utilizing B3LYP/6–311G(d,p)/PCM(H_2_O) (for details, see the Supporting Information) showed that the ^4^H_3_ conformation of α‐l‐*ido*‐aziridine **11** is greatly favored, with variations in the geometry about the C5−C7 bond (see the Supporting Information). Conversely, α‐l‐*idoA*‐aziridine showed both ^4^H_3_ and ^3^H_4_ (the latter 1.4 kcal mol^−1^ higher in energy) as relevant conformations. Interestingly, the ^2, 5^B boat conformation was also found as a relevant geometry for α‐l‐*idoA*‐aziridine, albeit with an energetic cost of 8.0 kcal mol^−1^. In addition, coupling constants (*J*) were calculated for the lowest‐energy conformations, and these were in excellent agreement with experimental NMR data (see the Supporting Information).

### Structural analysis of IDUA interactions with inhibitors 1–3

**Figure 4:**
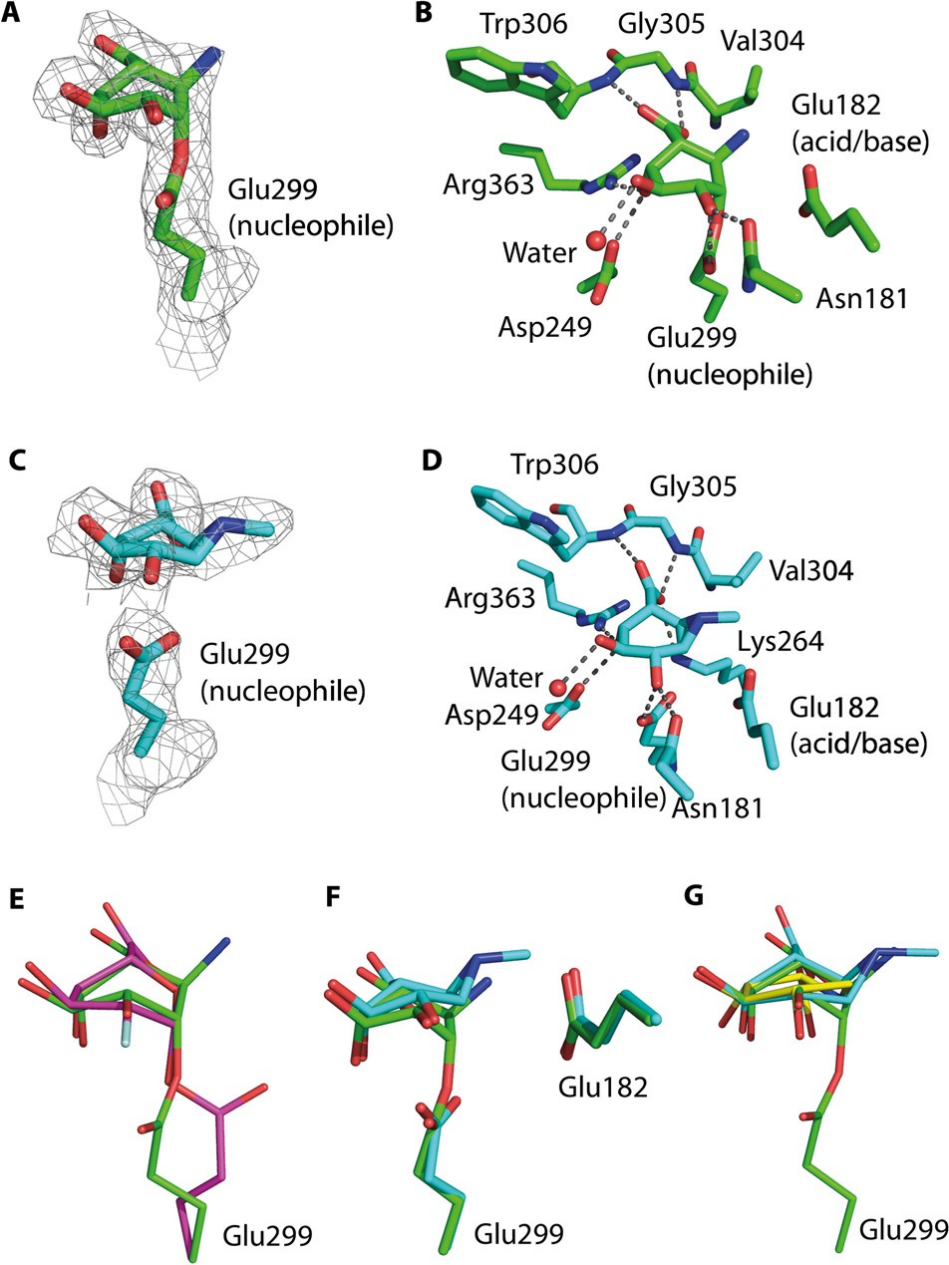
Structural insights into raIDUA complexed with ABPs. A) Structure of raIDUA complexed with a fragment of ABP **1**, which is covalently linked to the nucleophile Glu299. The maximum likelihood/*σ*_A_ weighted 2*F*_obs_−*F*_calc_ electron density map (gray) is contoured at 1.2 sigma. B) Structure of raIDUA covalently complexed with a fragment of ABP **1**, illustrating the active site residues that interact with the pseudo‐glycoside. C) Structure of raIDUA complexed with a fragment of ABP **3**. The nucleophile Glu299 is shown. The maximum likelihood/*σ*_A_ weighted 2*F*_obs_−*F*_calc_ electron density map (gray) is contoured at 1.0 sigma. D) Structure of raIDUA complexed with a fragment of ABP **3**, illustrating the active site residues that interact with the pseudo‐glycoside. E) Superposition of raIDUA covalently complexed with fragments of ABP **1** (green) and 2F‐IdoA (pink; PDB code 4KH2^8^). F) Superposition (based on alignment of protein main‐chain atoms) of raIDUA complexed with a fragment of ABP **1** (covalent, green) and a fragment of ABP **3** (transition state, cyan). G) Superposition (based on alignment of C3 and C4 atoms of each molecule) of raIDUA complexed with a fragment of ABP **1** (covalent, green), a fragment of ABP **3** (transition state, cyan), and IdoA‐DNJ (Michaelis complex, yellow; PDB code 4KGL).^8^

In order to study the mechanism of action of inhibitors **1**–**3**, we analyzed their conformations upon binding to IDUA by crystallographic studies. Compounds were applied in solution for various durations to raIDUA crystals (IDUA recombinantly expressed in the seeds of a *cgl* (complex glycan deficient) mutant of *Arabidopsis thaliana*; for details, see the Supporting Information). Data were collected from a crystal soaked with ABP **1** for 24 h to 2.02 Å resolution (Table S2), which revealed the structure of raIDUA in a covalent complex with **1** (Figure 4 A). The aziridine nitrogen is displaced by nucleophilic attack of the active site carboxylate to form a *trans*‐2‐amino ester (with the rest of the R group not visible in the electron density, presumably disordered due to its inherent flexibility; this region of the structure is exposed to the solvent). Interestingly, the pseudo‐glycoside was observed in a ^5^S_1_ skew‐boat conformation, which differs slightly from the distorted ^2, 5^B boat conformation reported for the previously described irreversible inhibitor 2‐deoxy‐2‐fluoro‐α‐l‐*ido*‐pyranosyl uronic acid (2F‐IdoA) covalently bound to IDUA.^8^ The observed ^5^S_1_ conformation of the covalent inhibitor **1**–enzyme complex supports predictions for the conformational itinerary followed by α‐iduronidase GH39 (Figure 1 A).^7^ The carboxylate group of the pseudo‐iduronic acid forms bidentate hydrogen bonds with the main‐chain nitrogen atoms of Gly305 and Trp306, the C4 hydroxyl group forms hydrogen bonds with Arg363 and Asp349, the C3 hydroxyl group interacts with Asp349 and a water molecule, and the C2 hydroxyl group forms hydrogen bonds with Asn181 and the nucleophile Glu299 (Figure 4 B).

In the covalent complex between IDUA and 2F‐IdoA, the nucleophile Glu299 is rotated by around 90° compared to the position observed in the complex here with the fragment of **1**,^8^ and the fluoro group at C2 may preclude an interaction with O*ϵ*2 of Glu299, causing it to rotate. However, the inhibitor **1**–IDUA complex presented here, bearing a hydroxyl group at C2 and showing an interaction with Glu299, is more likely to represent what occurs during catalysis (Figure 4 E). In an attempt to fully define the conformational inhibition of compounds **1**–**3**, raIDUA crystals were soaked with the ABPs for shorter durations. Data collected to 2.39 Å resolution (Table S2) on a crystal soaked with ABP **3** for 45 min revealed electron density in the active site of raIDUA consistent with the unreacted cyclophellitol aziridine **3** (Figure 4 C). A methyl group on the cyclophellitol aziridine was visible, but the rest of the R group was not evident and presumably disordered. Interestingly, the pseudo‐glycoside was observed in a ^2, 5^B conformation, which is the predicted transition state for GH39 α‐l‐iduronisase.^7^ The majority of the interactions with active site residues were the same as those described for the covalent complex with raIDUA (Figure 4 D), although a shift in position of the glycoside indicated that the carboxylate group additionally interacted with Lys264. The hydroxyl group at C2 forms a hydrogen bond with the nucleophile O*ϵ*2 of Glu299, but at a surprisingly short distance of 2.4 Å, suggesting a tight interaction. This close proximity results in a distance between the pseudo‐anomeric carbon and O*ϵ*1 of only 2.9 Å. These tight interactions, together with the ^2, 5^B conformation of the pseudo‐glycoside, suggest that we are observing the pseudo‐glycoside at the transition state; such structural observations are rare using wild‐type enzymes, but here it was possible due to the slow inactivation kinetics of **3**. The importance of the interaction between the glycoside and the C2 hydroxyl group supports work by others;^23^, ^24^ indeed, interactions at the 2‐position were estimated to contribute 18 kJ mol^−1^ binding energy to stabilization of the transition state for a β‐glucosidase during the glycosylation step of the catalysis.^23^ Based on this work, it was postulated that a hydrogen bond formed between the C2 hydroxyl group and the nucleophile would be optimal at the transition state, as the two groups come into close proximity during formation of the covalent glycosyl–enzyme bond.^23^

Superimposition of the main‐chain atoms for the two complexes revealed a shift in the position of the cyclophellitol aziridine to accommodate formation of the covalent bond (Figure 4 F). This engendered movement of between 0.2 and 0.4 Å at C5, C4, and C3, 0.5 Å at C2, 0.6 Å at the carbon at the position of the endocyclic oxygen, and 1.1 Å at the pseudo‐anomeric carbon. These structures, together with the previously reported structure of IDUA complexed with the inhibitor IdoA‐DNJ, in which the pseudo‐glycoside was observed in a ^2^S_0_ conformation (predicted Michaelis complex conformation), allow the full conformational itinerary for IDUA to be structurally defined. The structures of IDUA with IdoA‐DNJ (Michaelis complex) and **3** (transition state complex) and the fragment of ABP **1** (covalent complex) were overlapped at the C3 and C4 atoms (Figure 4 G). This clearly shows the electrophilic migration from the Michaelis complex in a ^2^S_0_ conformation, through the transition state in a ^2, 5^B conformation, to the covalent intermediate in a ^5^S_1_ conformation. At the pseudo‐anomeric carbon, there is a displacement of 0.74 Å on going from the Michaelis complex to the covalent intermediate. This is accompanied by a small (0.23 Å) movement at C2, but larger movement at C5 (0.72 Å) and the atom at the position of the endocyclic oxygen (0.70 Å), presumably to bring about the required migration at the anomeric position.

### rIDUA visualization by confocal fluorescence microscopy

**Figure 5:**
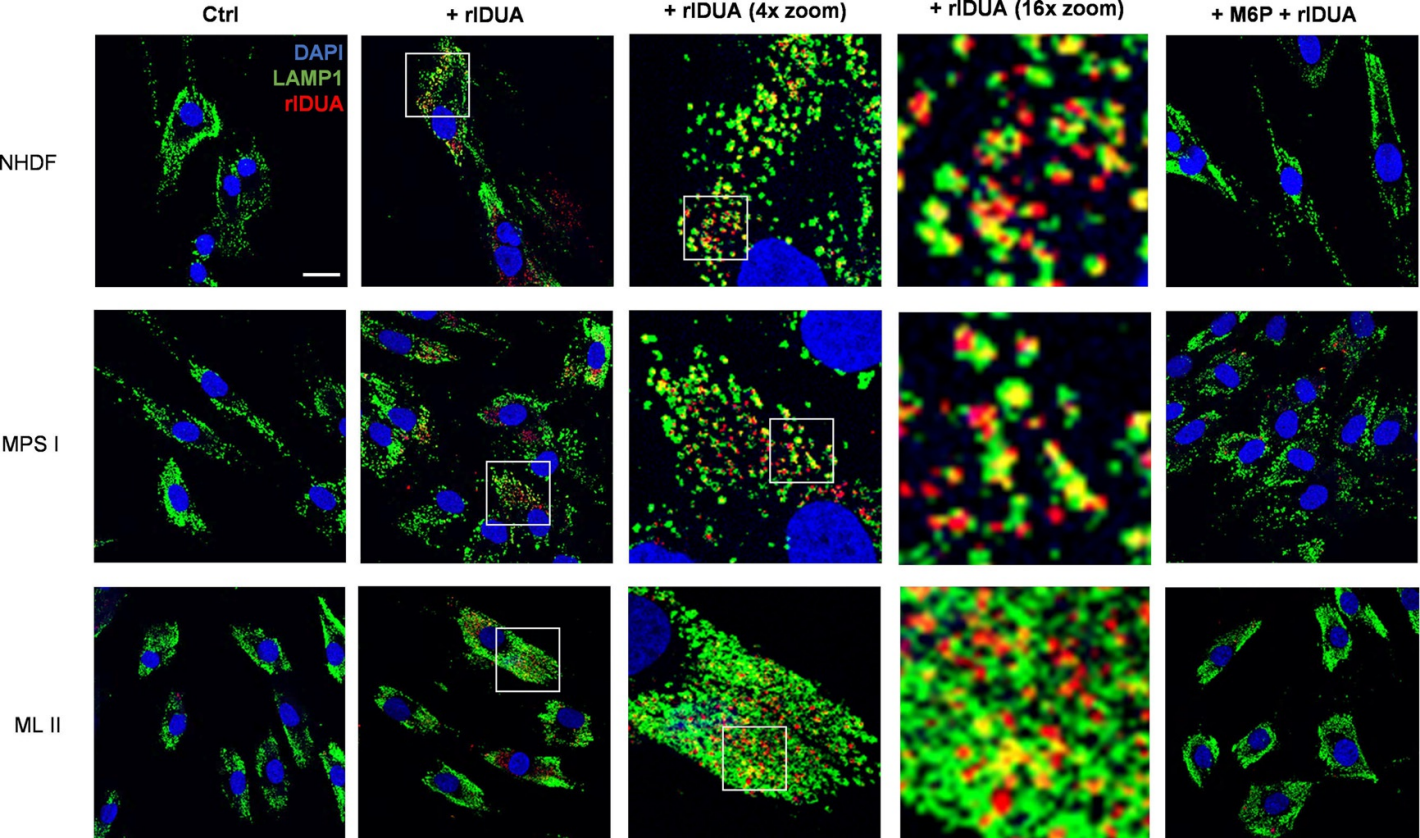
rIDUA visualization in human fibroblasts by confocal fluorescence microscopy. From top to bottom: NHDF, human normal dermal fibroblasts; MPS I, patient fibroblasts with mucopolysaccharidosis type I, and ML II, patient fibroblasts with mucolipidosis type II. From left to right: cells were incubated without (Ctrl) or with ABP **2**‐prelabeled rIDUA (+rIDUA, with successive zoomed‐in images from areas within the indicated white squares), or pre‐treated with mannose‐6‐phosphate prior to rIDUA incubation (+M6P+rIDUA). Color legend: nuclei were stained with DAPI (blue), lysosomes with immunostaining of lysosomal‐associated membrane protein 1 (LAMP1) (green), and rIDUA was labeled with ABP **2** (red). Scale bar=25 μm.

Finally, we investigated whether ABP **2** could be used to study rIDUA cellular uptake and lysosomal internalization. The majority of therapeutic glycosidases are modified with mannose 6‐phosphate (M6P) residues for their recognition by M6P receptors (MPRs) on the plasma membrane and consequent transport to the lysosomes. In order to track rIDUA within cells, NHDF and fibroblasts of MPS I and ML II patients were fed with pre‐labeled rIDUA‐ABP **2**, and the samples were analyzed by confocal fluorescence microscopy (Figure 5). After incubation for 16 h, points of fluorescence were observed within the lysosomes, indicating rIDUA lysosomal uptake by all of the analyzed fibroblast models. However, this was extinguished when the sample was pre‐incubated with M6P, which blocks M6P receptors (Figure 5, right). This result clearly indicates that rIDUA lysosomal uptake is mediated by MPRs,^25^ and that ABP **2** can be used to study the trafficking and localization of rIDUA within cells in MPS I and ML II patient cell lines.

## Conclusions

In conclusion, we have synthesized α‐l‐*ido*‐configured cyclophellitol aziridine, which is a key intermediate for generation of the first α‐l‐iduronidase ABPs. Key α‐l‐*ido*‐configured cyclohexene **8** could be an interesting starting point for the development of new IDUA inhibitors or chaperones. With the inhibitors described herein, we have conducted ABPP studies on rIDUA, showing that ABP **2** irreversibly labels rIDUA in a concentration‐ and time‐dependent manner, with optimum labeling at pH 4.5–5. We have further demonstrated covalent rIDUA inhibition by nano‐LC‐MS/MS, detecting a 7‐amino‐acid peptide fragment of rIDUA containing the nucleophilic residue bound to ABP **3**.

The conformations can strongly influence the inhibitory potencies and selectivities of cyclophellitol analogues.^26^ Inhibitors **1**–**3** are slower binders than the corresponding cyclophellitol aziridine conformers (such as β‐*gluco*‐aziridine ABP JJB367 for GBA^20^). DFT calculations have shown that inhibitors **1**–**3** adopt mainly a half‐chair conformation (^4^H_3_ and ^3^H_4_) in solution, with the ^2, 5^B conformation also as relevant geometry at an extra cost of 8.0 kcal mol^−1^. Crystallographic studies have shown that compounds **1**–**3** bind IDUA in a ^2, 5^B boat conformation in the Michaelis complex. The half‐chair conformations (^4^H_3_ and ^3^H_4_) of inhibitors **1**–**3**, as predicted by DFT calculations, differ from any of the reaction itinerary conformations of α‐l‐iduronidase depicted in Figure 1 A, and thus inhibitors **1**–**3** may need to overcome this 8.0 kcal mol^−1^ energetic barrier to adopt the Michaelis complex transition state (^2, 5^B) conformation. Therefore, we propose that the lower potency of α‐l‐*idoA*‐aziridine analogues is a manifestation of this energetic barrier. We have also provided structural evidence for covalent addition of the ABPs to the nucleophilic residue of raIDUA. In the process, we have defined the conformation of the cyclophellitol aziridine at the transition state and in the covalent intermediate, which supports predictions concerning the conformational itinerary followed by α‐l‐iduronidase. The insights gained through these studies should help in the design of closer conformational ^2, 5^B analogues by the use of different electrophilic traps or reactive species for the generation of improved inhibitors or molecular chaperones, with the end goal being the future provision of improved therapies for MPS I patients. Although IDUA labeling with ABP **2** in complex biological samples was not successful due to the lower potency of this analogue compared with previously reported glycosidase probes, we have demonstrated that ABP **2** can be used to study the localization and trafficking of rIDUA within cultured cells, and have shown that the rIDUA–ABP **2** complex is recognized by MPRs and internalized in lysosomes.

## Experimental Section

See the Supporting Information for experimental details.

## Conflict of interest

The authors declare no conflict of interest.

## Supporting information

As a service to our authors and readers, this journal provides supporting information supplied by the authors. Such materials are peer reviewed and may be re‐organized for online delivery, but are not copy‐edited or typeset. Technical support issues arising from supporting information (other than missing files) should be addressed to the authors.

Supplementary
