# Supplementary material for: New Irreversible α‐l‐Iduronidase Inhibitors and Activity‐Based Probes
Source: Chemistry. 2018 Nov 26;24(71):19081–8. doi: 10.1002/chem.201804662 (PMC6343074; doi:10.1002/chem.201804662)
Supplement: Supplementary file 1 — Supplementary [file CHEM-24-19081-s001.pdf]

CHEMISTRY
A European Journal

Supporting Information

New Irreversible α-L-Iduronidase Inhibitors and Activity-Based Probes

Marta Artola+,*[a] Chi-Lin Kuo+,[b] Stephen A. McMahon,[c] Verena Oehler,[c] Thomas Hansen,[a] Martijn van der Lienden,[b] Xu He,[d] Hans van den Elst,[a] Bogdan I. Florea,[a] Allison R. Kermode,[d] Gijsbert A. van der Marel,[a] Tracey M. Gloster,[c] Jeroen D. C. Codée,[a] Herman S. Overkleeft,[a] and Johannes M. F. G. Aerts*[b]

chem_201804662_sm_miscellaneous_information.pdf

Electronic Supplementary Information

New irreversible α-L-iduronidase inhibitors and activity-based probes

Marta Artola,*[,¶,a] Chi-Lin Kuo,[¶,b] Stephen A. McMahon,[c] Verena Oehler,[c] Thomas Hansen,[a] Martijn van der Lienden,[b] Xu He,[d] Hans van den Elst,[a] Bogdan I. Florea,[a] Allison R. Kermode,[d] Gijsbert A. van der Marel,[a] Tracey M. Gloster,[c] Jeroen D. C. Codée,[a] Herman S. Overkleeft,[a] Johannes M. F. G. Aerts*[b]

*Corresponding authors: m.e.artola@lic.leidenuniv.nl; j.m.f.g.aerts@lic.leidenuniv.nl

[a]*Department of Bio-organic Synthesis, Leiden Institute of Chemistry, Leiden University, Einsteinweg 55, 2333 CC Leiden, The Netherlands.*

[b]*Department of Medical Biochemistry, Leiden Institute of Chemistry. Einsteinweg 55, 2333 CC Leiden, The Netherlands.*

[c]*School of Biology, Biomedical Sciences Research Complex, University of St Andrews, North Haugh, St Andrews, Fife, KY16 9ST, UK.*

[d]*Department of Biological Sciences, Simon Fraser University, 8888 University Drive, Burnaby, BC V5A 1S6, Canada.*

[¶]*These authors share first authorship of this work*

TABLE OF CONTENTS

1. Supporting Figures and Tables S2
2. Materials and Methods S7
   2.1. Biochemical and Biological Methods S7
   2.2. DFT Calculations S11
   2.3. Chemical Synthesis ` S15
      2.3.1. General Experimental Details S15
      2.3.2. Synthesis and Characterization Data of Compounds 1–3 S16
3. NMR Spectra S22
4. References S32

1. Supporting Figures

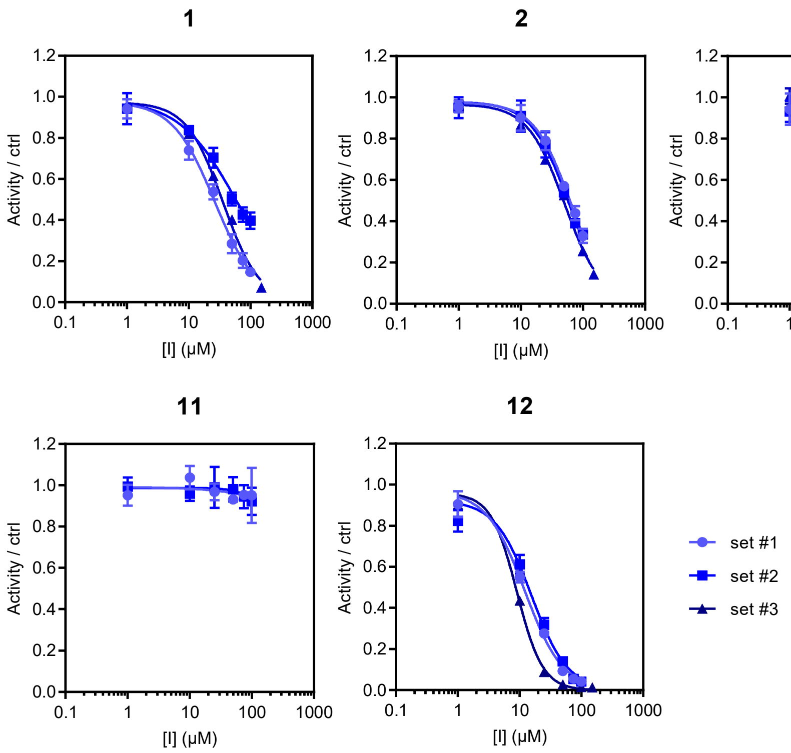

Compd	IC_{50} (µM), n =3 ± SD
**1**	40.6 ± 17.0
**2**	58.1 ± 6.66
**3**	65.5 ± 5.73
**11**	> 100
**12**	12.2 ± 3.24

Figure S1. *In vitro* inhibition of recombinant α-L-iduronidase (rIDUA) by compounds **1**-**3**, **11** and **12** at 1 hour incubation time (compounds **1**-**3**, **12** = triplicate sets of experiment, each with technical triplicates; **11** = duplicate sets of experiment). Y-axis depicts measured 4-MU-α-IdoA activity normalized to the measured activity for the control group treated with DMSO. X-axis depicts inhibitor concentrations. Error range in inhibition curves = SD from technical triplicates.

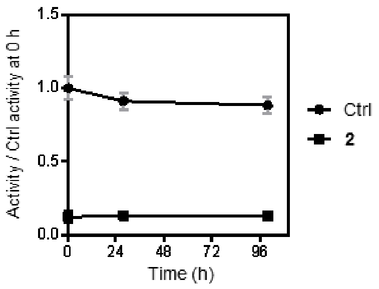

Figure S2. Irreversible inhibition of rIDUA by ABP **2**. rIDUA remains inactivated by ABP **2** for at least 100 h after unbound ABP **2** was removed from the samples. Error range = SD from technical triplicates.

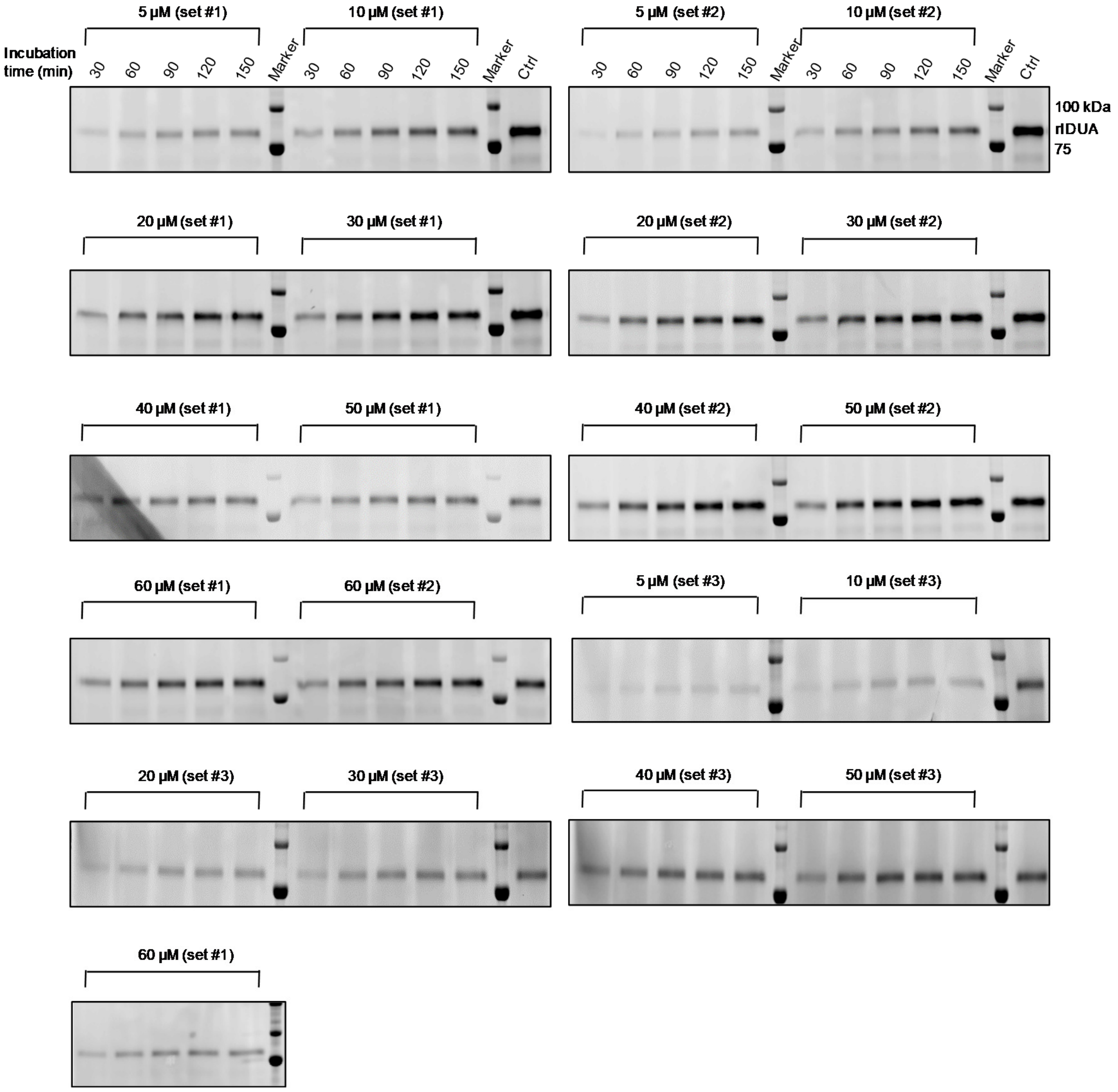

Figure S3. SDS-PAGE gels used for labelling kinetic studies of ABP **2** with rIDUA. rIDUA was labelled at different incubation time periods (30-150 min) and at different ABP **2** concentrations (5-60 µM), before SDS-PAGE and fluorescent detection and quantification. The labelling experiment was performed in triplicate sets.

	**Specific activity** (nmol h^{-1})		**Calculated enzyme amount** (fmol)
	Recombinant enzyme (per 100 fmol)	NHDF lysate (per 10 µg)	NHDF lysate (per 10 µg)
IDUA	2.44 ± 0.11	0.40 ± 0.01	16.39
GBA	8.62 ± 1.26	2.12 ± 0.10	24.59
GBA/ IDUA	***3.53***	***5.3***	***1.50***

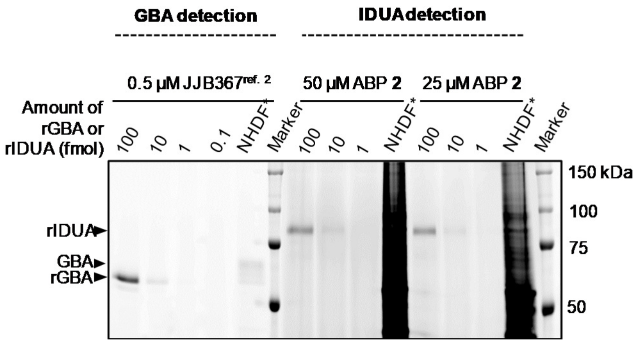

Figure S4. Detection of endogenous IDUA in human fibroblsts (NHDF) lysates. A) Comparing endogenous enzyme amount in human fibroblasts (NHDF) lysates for IDUA *vs* glucocerebrosidase (GBA), by comparing measured specific activity presented in lysates *vs* from known amount of rIDUA or rGBA with 4-methylumbelliferyl-glycoside substrates. B) Comparing ABP labelling of known amounts of rGBA and rIDUA vs ABP labelling in NHDF lysates. *10 µg protein was loaded.

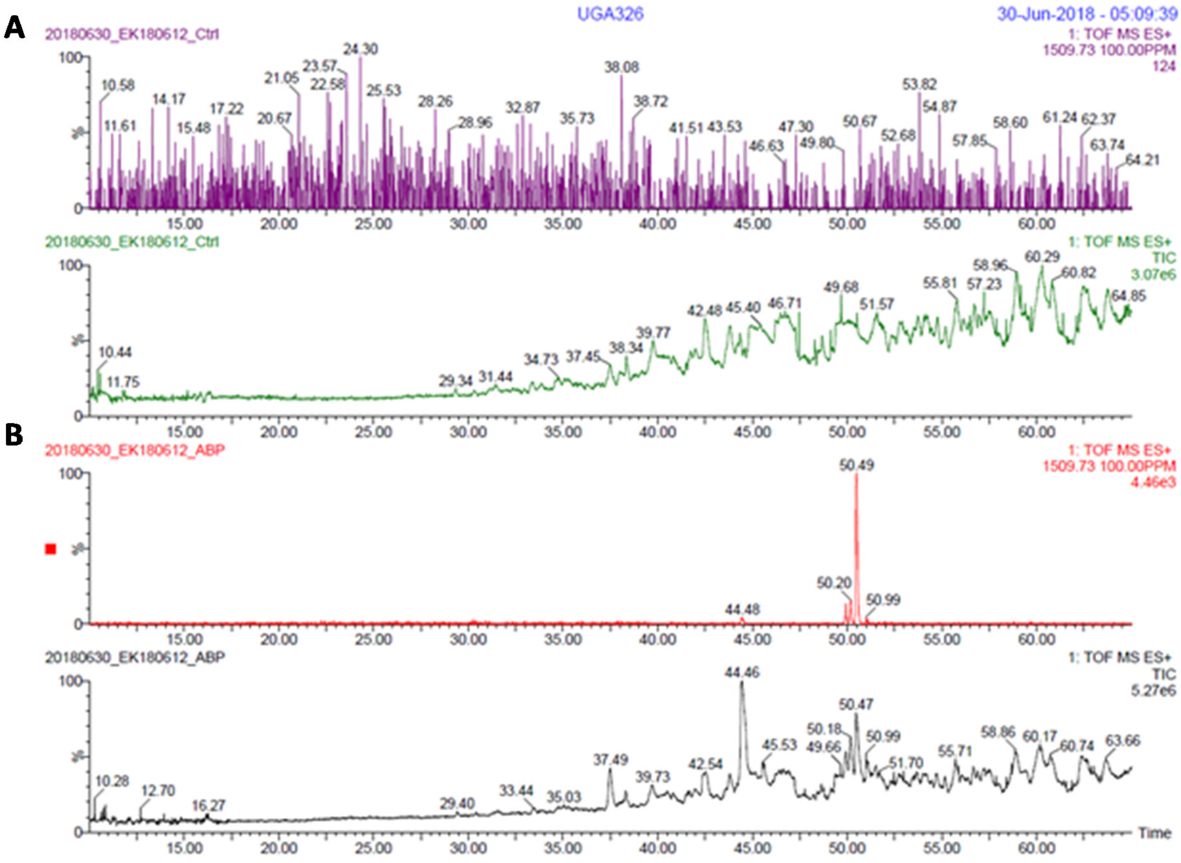

Figure S5. Chromatogram of sample containing rIDUA active site peptide labelled without (Ctrl) or with ABP **3**.

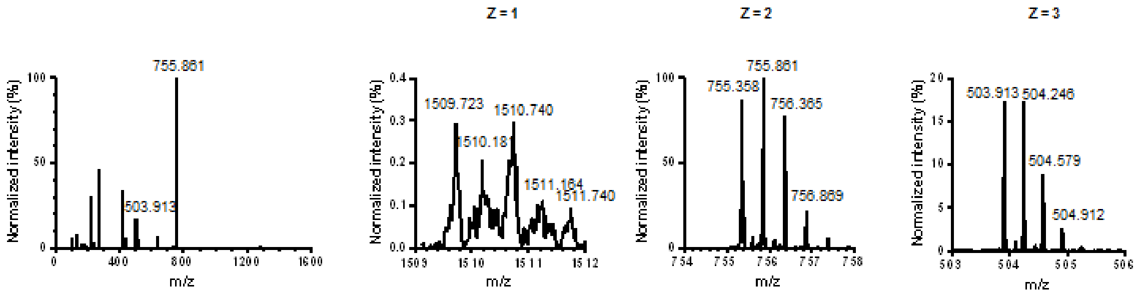

Figure S6. Mass spectrum of sample containing rIDUA active site peptide labelled with ABP **3**.

Table S1. Theoretical and experimental m/z values for rIDUA active site peptide labelled with ABP **3**.

z	Theoretical		Experimental		Delta	ppm	
1	1509.725	1510.729	1509.723	1510.740	1.017	1.32	7.28
2	755.3663	755.868	755.3578	755.8612	0.5034	11.25	9.00
3	503.9133	504.2478	503.9127	504.2455	0.3328	1.19	4.56

Table S2. Data collection and refinement statistics for raIDUA in complex with fragments of **1** and **3**.

	raIDUA in covalent complex with fragment of **1** (PDB: 6I6R)	raIDUA in non-covalent complex with fragment of **3** (PDB: 6I6X)
**Data collection**		
Space group	$P2_12_12_1$	*H*3
Cell dimensions		
a, *b*, *c* (Å)	206.9, 69.9, 93.7	259.2, 259.2, 71.0
α, β, γ (°)	90.0, 90.0, 90.0	90.0, 90.0, 120.0
Resolution (Å)	66.25-2.02 (2.05-2.02)	29.73-2.39 (2.44-2.39)*
R_{merge}	0.164 (1.047)	0.124 (0.866)
R_{pim}	0.080 (0.50)	0.061 (0.433)
$I / \sigma I$	5.4 (1.3)	10.2 (1.3)
Completeness (%)	94.4 (97.8)	99.6 (94.0)
Redundancy	4.9 (5.0)	5.2 (4.8)
$CC_{1/2}$	0.99 (0.54)	0.995 (0.669)
**Refinement**		
Resolution (Å)	66.25-2.02	29.73-2.39
No. reflections	80558	66351
R_{work} / R_{free}	0.21 / 0.24	0.21 / 0.23
No. atoms		
Protein	9658	9818
Ligand	26	14
Water	250	294
B-factors		
Protein	38.6	43.2
Ligand	45.3	59.8
Water	35.2	39.2
Overall		
R.M.S. deviations		
Bond lengths (Å)	0.012	0.010
Bond angles (°)	1.56	1.45

*Values in parentheses are for highest-resolution shell.

2. Materials and Methods

2.1. Biochemical and Biological Methods

Materials

Recombinant human iduronidase (rIDUA) was obtained from Genzyme (Aldurazyme). Normal human dermal fibroblasts (NHDF) were obtained from Lonza. Human patient fibroblasts (MPS-I and ML-II) were obtained with consent from donors from the Academic Medical Center in Amsterdam, the Netherlands. 4-methylumbelliferyl α-L-iduronide (4-MU-α-L-IdoA) was purchased from Glycosynth. Pierce^{TM} Bicinchoninic acid (BCA) protein assay kit and Pierce™ Polyacrylamide Spin Desalting Columns 7K MWCO was acquired from Thermo Fisher Scientific. All other chemicals were obtained from commercial sources.

Cell culture and lysate preparation

Fibroblasts were cultured in Dulbecco's modified Eagle's medium: Nutrient Mixture F-12 (DMEM/F-12, Invitrogen), containing 10% (v/v) heat-inactivated fetal calf serum, 200 µg/mL penicillin, 200 µg/mL streptomycin, at 37 °C and 5% CO_2. Confluent Fibroblasts were subcultured at a ratio of 1:4 each week. To prepare lysates, cells were washed three times with phosphate-buffered saline (PBS), detached by scraping in the presence of ice-cold lysis buffer (25 mM K_2HPO_4/KH_2PO_4 pH 6.5, supplemented with 0.1% (v/v) Triton X-100 and protease inhibitor cocktail tablet Roche (version 12)), and collected in Eppendorf tubes. The collected suspension was vortexed vigorously, incubated on ice for 1 h, freeze-thawed once using liquid nitrogen, and stored at -80 °C. Concentration of lysates was determined using BCA kit.

Iduronidase activity assay using 4-MU-α-L-IdoA as substrate

Samples containing enzyme were diluted in assay buffer (150 mM citrate/Na_2HPO_4, pH 4.5, supplemented with 0.1% (v/v) Triton X-100, 0.15 M NaCl, 33 mM $CaCl_2$, 33 mM $MgCl_2$), and load onto a black flat-bottom 96-well plate (Greiner) in triplicates of 25 µL per well. Negative controls (blank) were prepared by substituting enzyme samples with buffer. For reaction, samples were added with 25 µL of 360 µM 4-MU-α-L-IdoA[1] (prepared in assay buffer supplemented with 0.1 % (w/v) bovine serum albumin (BSA)), and incubated at 37°C for 30 min to 4h. Reaction was stopped by adding 200 µL 1M Glycine-NaOH (pH 10.3), and fluorescence was measured using a LS-55 fluorometer (PerkinElmer) at λ_{ex} = 366 nm and λ_{em} = 445 nm.

Apparent IC_{50} values of inhibitors and ABPs on recombinant human iduronidase and cell lysates

Inhibitors and ABPs were diluted at various concentrations in 12.5 µL assay buffer and incubated with 12.5 µL rIDUA (5.8 ng, or 70 fmol) or fibroblast lysates (50 µg protein), both diluted in assay buffer, at 37°C for 1 h (rIDUA) or 4 h (fibroblasts lysates), at 1% (v/v) DMSO concentration. This was followed by iduronidase activity assay (30 min incubation time) described in the previous section. The detected 4-MU fluorescence at each concentration for each compound were normalized to the fluorescence from the control sample without inhibitor, and data were fitted with [inhibitor] *vs* response – variable slope (four parameters) function in Graphpad Prism 7.0 software to obtain apparent IC_{50} values.

Labelling and SDS-PAGE of recombinant human iduronidase

To prepare for labelling, rIDUA stock (0.58 µg/µL in PBS) was diluted with assay buffer to final concentration of 10 ng (120 fmol) in 14 µL assay buffer, if not otherwise specified. ABP **2** stock (15 mM in DMSO) was diluted subsequently with DMSO and then assay buffer, to 8x of intended assay concentration obtaining 4% (v/v) DMSO. For labelling, 2 µL of ABP **2** dilution was incubated with 14 µL enzyme dilution at 37°C for the intended time periods. Labelling was terminated by denaturing samples with 4 µL sample buffer (5x Laemmli buffer, containing

0.3M Tris-HCl pH 6.8, 50% (v/v) 100% glycerol, 8% (w/v) Dithiothreitol (DTT), 10% (w/v) sodium dodecyl sulfate (SDS), 0.01% (w/v) bromophenol blue) and heated at 98°C for 5 minutes. SDS-PAGE and fluorescence detection procedures followed the previously described methods.[2]

Labelling of recombinant human iduronidase at different pH

Assay buffers with pH 2.5 – 8.0 were used to dilute rIDUA and ABP **2**. The experiment was performed with 10 ng rIDUA at 10 µM ABP **2** (assay concentration) in 16 µL volume and 1 h incubation time, followed by denaturation, SDS-PAGE, and fluorescence scan.

Competitive ABPP experiments

For the competitive ABPP (cABPP) experiment using 4MU-α-IdoA as an active-site directed inhibitor, 10 ng rIDUA, or no-enzyme blank (PBS), was prepared in 7 µL assay buffer (pH 4.5). This was pre-incubated with 7 µL assay buffer containing 4MU-α-IdoA (0.2 -20 mM) on ice for 5 min, then incubated with 1 µL ABP **2** (750 µM, 8 % (v/v) in assay buffer) or DMSO control (8 % (v/v) in assay buffer) at 37°C for 2 h. In the same experiment, 7 µL SDS (4 % (w/v)) was added to the 7 µL enzyme, pre-incubated at 98°C for 2 min, and incubated with ABP **2** at 37°C for 2 h. For the cABPP experiment with compound **1**, 10 ng rIDUA was prepared in 12 µL assay buffer, pre-incubated with 2 µL compound **1** (2.8 mM – 70 µM, 18.6% DMSO in assay buffer) at 37°C for 2 h, then incubated with 2 µL ABP **2** (100 µM, 4 % DMSO in assay buffer) at 37°C for 2 h. Samples were then denatured, and subjected to SDS-PAGE and fluorescence scan.

Reversibility of labelling of ABP 2 on recombinant human iduronidase

5 µL rIDUA stock (0.58 µg/µL in PBS) was diluted with 20 µL assay buffer without Triton X-100, and incubated with either 25 µL ABP **2** (150 µM and 1 % (v/v) DMSO) or 25 µL assay buffer containing 0.1% (w/v) DMSO for 1 h at 37°C. Thereafter, 45 µL of sample was passed through a desalting column, and diluted with 216 µL assay buffer (with Triton X-100) to a final enzyme concentration of 1 ng/µL. For assessing the reversibility of labelling/inactivation of ABP **2** on rIDUA, both samples were kept at 4 °C until subjecting to iduronidase activity assay using 4-MU-α-IdoA at the following time points after desalting: 0.5, 28, and 100 h. Iduronidase activity assay was performed with 1 µL sample in triplicates, and assay buffer was used as blank (Figure S2).

Kinetic parameters of ABP 2

The kinetic parameters of ABP **2** for rIDUA was determined by a SDS-PAGE-based assay, in which the intensity of fluorescent signal from ABP **2**-labelled rIDUA on the wet slab gel is proportional to the extent of rIDUA inactivation, when compared to the signal from a control group with saturate labelled rIDUA. The experiment was performed by labelling rIDUA with ABP **2** for different time periods and at different ABP **2** concentrations (which were >> enzyme concentration), and the kinetic parameters k_{inact} (pseudo first-order inactivation rate constant) and K_I (inhibition constant) were obtained using non-linear curve fitting. Briefly, rIDUA was diluted in a series of 1.5 mL Eppdendorf tubes to 10 ng (120 fmol) per 14 µL, and labelled with 2 µL ABP **2** (diluted in DMSO and assay buffer to various concentrations and [DMSO] fixed at 8% (v/v)) for 30, 60, 90, 120, or 150 min. Concentrations in assay were 5 µM, 10 µM, 20 µM, 30 µM, 40 µM, 50 µM, or 60 µM for ABP **2**, 7.5 nM for rIDUA, and 1 % (v/v) for DMSO. Reaction was terminated by incubating samples with sample buffer at 98°C for 5 min. Samples were subjected to SDS-PAGE and fluorescence scan, and Cy5 fluorescence from the ABP **2**-labelled rIDUA was quantified using ImageQuant software (GE Healthcare). The incubation condition for maximum labelling (complete inactivation) on rIDUA for ABP **2** was determined to be 60 µM and 150 min, and a control sample with this condition was loaded on every gel to allow normalization for samples in the same gel. After normalization, the average values at each ABP **2** concentration from n =3 sets of experiment were plotted in a time *vs* % labelling (inactivation) graph, and the plotted data were fitted with one-phase exponential association function to obtain the rate constant k at each ABP **2** concentration. Finally, the obtained k values were plotted

against ABP **2** concentrations, and the data were fitted with a Michaelis-Menten equation to obtain k_{inact} and K_I values for ABP **2** on rIDUA. All non-linear curve-fitting was performed using Graphpad Prism 7.0 software.

ABP 2 labelling in fibroblast lysates

Fibroblast lysates (40 µg protein) were diluted in assay buffer and incubated with ABP **2** at final concentrations of 1, 2, 5, or 10 µM (1% (v/v) DMSO) at 37°C for 4 h. Samples were denatured and subjected to SDS-PAGE, and wet slab gel was scanned for Cy5 fluorescence and then stained with Coomassie Brilliant Blue solution for assessing total protein amounts in each lane of sample.

Fibroblasts uptake of ABP 2-labelled recombinant human iduronidase

rIDUA was labelled with ABP **2** at 75 µM for 1 h at 37 °C, and unbound ABP **2** in solution was removed followed the described procedures in an earlier section. The labelled rIDUA were diluted in assay buffer without Triton X-100, to a concentration of 10 ng/µL. For the uptake experiment, human normal and patient fibroblasts were sub-cultured 1 day before treatment in 12-well plates (1 mL culture medium per well) with or without glass coverslips. Cells were then pre-treated with 4 mM mannose-6-phosphate for 1 h, followed by treating with ABP **2**-labelled rIDUA (100 ng/mL culture medium) for 20 h. A control group was included for each cell type, treated only with water and assay buffer. Confocal microscopy analysis was carried out following a previously described procedure[3], where samples were fixed with 4% formaldehyde, permeabilized with 0.1% (w/v) saponin and 2% (w/v) BSA, and immuno-stained for the lysosomal membrane protein LAMP-1 using mouse anti human LAMP1 (Southern Biotech) as primary antibody and donkey anti mouse Alexa488 (Molecular Probes) as secondary antibody. The coverslips were mounted to microscopy slides using ProLong™ Diamond Antifade Mountant with DAPI (Thermo Fisher), and scanned using a Leica SP8 confocal microscope for DAPI, Alexa488, and Cy5 fluorescence with a 40x oil-immersed objective. Pictures at each fluorescence channel were captured at 1024 x 1024 resolution, with *n* =3 frame averages.

Comparing specific activity and ABP labelling between GBA and IDUA

For measurement of GBA activity, 100 fmol of rGBA (Cerezyme/Imiglucerase, Genzyme) or NHDF lysates (10 µg protein) were diluted in 25 µL of GBA buffer (150 mM Citric acid/Na_2HPO_4, 0.1 % (v/v) (Triton X-100), 0.2 % (w/v) Sodium Taurocholate) and incubated with 100 µL GBA substrate mixture (3.75 mM 4-MU-β-D-glucopyranoside, Glycosynth, in GBA buffer, 0.1 % (w/v) BSA) for 30 min at 37°C. IDUA activity was measured following the methods described in previous section. For ABP labelling of GBA, rGBA (100 – 0.1 fmol) or NHDF lysates (10 µg protein) were diluted in 14 µL GBA buffer, and incubated with 0.5 µM JJB367[2] for 30 min at 37°C ([DMSO] = 0.5 % (v/v)). For ABP labelling of IDUA, rIDUA (100 – 1 fmol) or NHDF lysates (10 µg protein) were diluted in 14 µL assay buffer and incubated with either 50 µM or 25 µM ABP **2** for 4 h at 37°C ([DMSO] = 0.5 % (v/v)). Samples were denatured, and proceeded to SDS-PAGE and fluorescent detection.

LC-MS/MS identification of rIDUA active site peptide

A total of 10 µg rIDUA was diluted in assay buffer and incubated with either 75 µM ABP **3** or DMSO (negative control) for 1 h at 37°C in 100 µL volume ([DMSO] = 0.5 % (v/v)). The samples were then added with 100 µg BSA (100 µL), and followed by chloroform/methanol precipitation and reduction/alkylation procedures described previously.[4] Consequently, the samples were dissolved in 2 % (w/v) SDS and diluted with 50 mM Tris-HCL (pH 7.8) to a final SDS concentration of 0.005 % (w/v). The samples were then concentrated with size exclusion columns (Amicon 10k) to a volume of 74 µL, and digested O/N at 25 ºC with 1.1 µg Chymotrypsin (Promega) in the presence of 10 mM $CaCl_2$. Digested peptides were pulled-down using 50 µL of Streptavidin paramagnetic beads (MyOne T1, ThermoFisher) in 1 mL of pull-down buffer (50 mM Tris-HCL (pH 7.5), 150 mM NaCl, 0.5 % (w/v) SDS) for 1 h at RT under vigorous shaking. The beads were washed stringently following previously described procedures[4] and eluted with 100 µL of elution buffer (25 % (v/v) Acetonitrile, 5 % (v/v) formic acid, 70

% H_2O, 10 µM biotin) for 30 min at 37°C. Afterwards, acetonitrile in the supernatant were evaporated using a Speedvac at 45°C, and this was followed by desalting using StageTips. The eluate were evaporated and reconstituted in 20 µL of LC-MS sample solution (95:3:0.1, H_2O:acetonitrile:formic acid) for LC-MS/MS analysis. Peptide samples were analysed with a two hour gradient of 5% to 25% acetonitrile on nano-LC, hyphenated to an LTQ Orbitrap and identified by manual search for the theoretical m/z values of the active site peptide and its MS/MS fragments labelled with ABP **3**

Recombinant expression and purification of IDUA in seeds of *Arabidopsis thaliana*

Recombinant human α-L-iduronidase (abbreviated to raIDUA to distinguish from rIDUA obtained from Genzyme) was produced in seeds of *Arabidopsis thaliana cgl* (***c***omplex ***gl***ycan deficient) line A4.7[5] in which the seeds (T3 generation) accumulated raIDUA to 7.2 ± 0.6 % total soluble protein (9.8 µg/mg dry seeds). raIDUA was purified to homogeneity from the T3 seeds using concanavalin A-sepharose and anti-IDUA affinity chromatography as described previously[6,7]. In human IDUA, there are six N-linked glycosylation sites. The oligosaccharide structures at each site of rIDUA secreted from a Chinese hamster ovary (CHO) cell line have been determined by mass spectrometry[8]. The raIDUA expressed in the seeds of the *cgl* mutant of *Arabidopsis* has much reduced complexity in these N-linked glycans, the majority of which are non-matured, high mannose N-glycans[5,9].

Crystallization of raIDUA

raIDUA was further purified by size exclusion chromatography using an S200 10/300 column (GE Healthcare) equilibrated in 20 mM Tris, pH 7.0, 500 mM NaCl, and 0.02 % sodium azide. The fractions containing raIDUA were buffer exchanged using a PD10 desalt column (GE Healthcare) into 20 mM dimethylglutaric acid, pH 6.0, 0.2 M NaCl, 5% (v/v) glycerol, and 5% (v/v) ethanol and concentrated to 10 mg/ml for crystallization. Crystallization was performed in a 24 well plate using hanging-drop vapour diffusion. The rhomboid-plate shaped crystals grew at room temperature from 0.1 M HEPES, pH 7.5, 0.26 M sodium potassium tartrate, 20% (w/v) polyethylene glycol 3350 (optimized from the crystallization condition reported by Bie *et al.*[10]). Crystals were soaked in mother liquor containing a minute amount of solid **1** for 24 hours or **3** for 45 minutes, before being harvested. Crystals were cryo-protected in a solution containing the mother liquor plus 30% glycerol prior to vitrification in liquid nitrogen.

Data collection and processing for raIDUA crystals

X-ray diffraction data were collected at Diamond Light Source (DLS) on beamlines I03 and I04; the data processing and refinement statistics can be found in Table S2. Diffraction data were processed either using the FastDP pipeline[11] (which utilises XDS[12] with Aimless[13]) or Xia2[14] (also with XDS[12] with Aimless[13]). Molecular replacement was performed using Phaser[15] with Protein Data Bank (PDB) entry 4JXO as the search model. Refinement was performed using REFMAC5[16] and manual model building was done using Coot[17]. Structures were validated using PDB_REDO[18]. Models for the fragments of **1** and **3** were built in JSME[19] and the libraries generated with PRODRG[20].

2.2 DFT Calculations

Geometry optimization

By using the conformer distribution search option included in the Spartan 14 program[21], exclusively the $^{4}H_{3}$ conformation of the structure was found. Only notable variations of the geometry were found at the C5-C7 bond, including multiple rotamers which were significant higher in terms of energy.

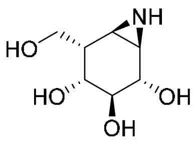 α-L-*Ido* **11**	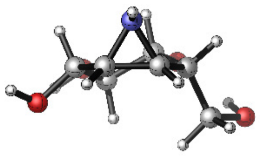	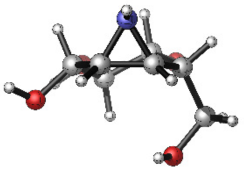	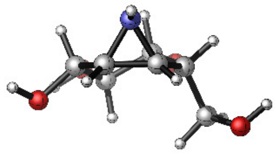
	$^{4}H_{3}$ ***tg***	$^{4}H_{3}$ ***gg***	$^{4}H_{3}$ ***gt***
ΔG_{aq}^{T} **(kcal/mol)**	0.0	1.5	1.6
**Geometry**	D1: -45.4 ° D3: -49.6 ° D5: -2.3 °	D1: -43.4 ° D3: -49.8 ° D5: -3.1 °	D1: -45.6 ° D3: -49.5 ° D5: -3.0 °

For α-L-*idoA*-aziridine the $^{4}H_{3}$ conformation was also found as lowest energy conformer, but in this case the $^{3}H_{4}$ was only 1.4 kcal/mol higher in terms of energy.

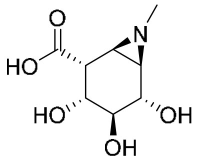 α-L-*IdoA*	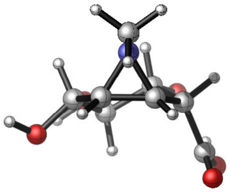	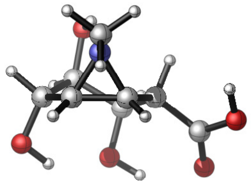	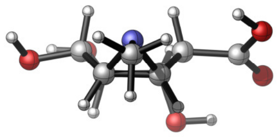
	$^{4}H_{3}$	$^{3}H_{4}$	$^{2,5}B$
ΔG_{aq}^{T} **(kcal/mol)**	0.0	1.4	8.0
**Geometry**	D1: -44.1 ° D3: -53.5 ° D5: -2.3 °	D1: 51.7 ° D3: 43.8 ° D5: 0.9 °	D1: -52.1 ° D3: 48.0 ° D5: 2.0 °

All calculations were performed with DFT as level of theory in combination with the B3LYP hybrid functional. A conformer distribution search option included in the Spartan 14 program[21], in gas-phase with the use of 6-31G(d) as basis set, was used as starting point for the geometry optimization. All generated structures were further optimized with Gaussian 09[22] at 6-311G(d,p). Optimization was done in gas-phase and subsequently corrections for solvent effects were done by the use of a polarizable continuum model using water as solvent parameter. The free Gibbs energy of the computed conformations was calculated using Equation (1) in which ΔE_{gas} is the gas-phase energy (electronic energy), ΔG_{RRHO}^{T} (T= 298.15 K and pressure= 1 atm.) is the sum of corrections from

the electronic energy to free Gibbs energy in the rigid-rotor-harmonic-oscillator approximation (RRHO) also including zero-point-vibrational energy, and ΔG^{T}_{solv} is their corresponding free solvation Gibbs energy.

$$\begin{aligned}\Delta G^{T}_{aq} &= \Delta E_{gas} + \Delta G^{T}_{gas,RRHO} + \Delta G_{solv} \\ &= \Delta G^{T}_{gas} + \Delta G_{solv}\end{aligned} \qquad (1)$$

The used free energies include unscaled zero-point vibrational energies. Visualisation of the conformations of interest was done with CYLview.[23]

NMR calculations

Based on the optimized lowest energy structure the spin-spin coupling constants were calculated according to the work of Rablen and Bally[24] with the use of 6-311g(d,p) u+1s as basis set and PCM(H_2O) as solvent model and a scaling factor of 0.92. The calculated total nuclear spin-spin coupling terms were used as calculated spin-spin coupling constants.

Table S3. Experimental coupling constants of α-L-idose configured cyclophellitol aziridine **11** compared to DFT calculated coupling constants.

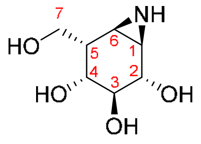

11
α-L-Ido

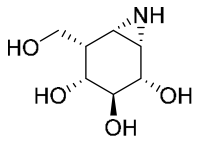

β-L-Ido

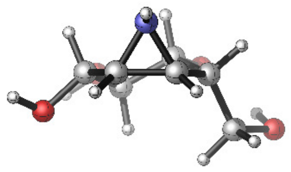

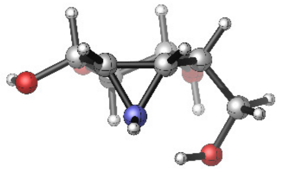

H-H Coupling	Exp. $^3J_{(H,H)}$ (Hz)	α-L-*ido* DFT calc. $^3J_{(H,H)}$ (Hz)	β-L-*ido* DFT calc. $^3J_{(H,H)}$ (Hz)
H1-H6	6.0	5.8	6.8
H1-H2	n.d.	3.1	2.9
H2-H3	7.4	7.5	8.2
H3-H4	10.5	10.2	9.3
H4-H5	5.6	6.3	9.8
H5-H6	1.6	1.3	5.9
H5-H7a	4.4	2.8	0.9
H5-H7b	11.1	10.3	4.3

Coupling constants were determined by ^{1}H NMR experiments (exp.). n.d.: values not determine due to very small coupling constant (J < 1 Hz).

Table S4. Experimental coupling constants of α-iduronic acid configured cyclophellitol aziridine **1** compared to DFT calculated coupling constants of the methylated α-iduronic configured cyclophellitol aziridine.

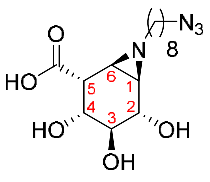

1

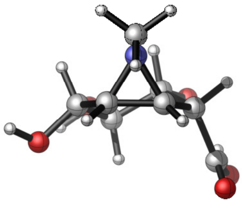

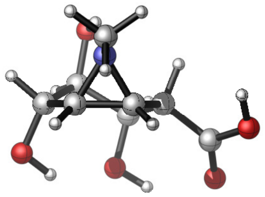

H-H Coupling	Exp. $^3J_{(H,H)}$ (Hz)	α-L-*idoA* DFT calc. $^3J_{(H,H)}$ (Hz) $^4H_3$ (0.0 kcal/mol)	α-L-*idoA* DFT calc. $^3J_{(H,H)}$ (Hz) $^3H_4$ (1.4 kcal/mol)	α-L-*idoA* Boltzmann-weighted average $^3J_{(H,H)}$ (Hz)
H1-H6	n.d.	6.0	6.2	6.0
H1-H2	-	0.0	1.7	0.3
H2-H3	6.8	7.2	2.9	6.4
H3-H4	9.2-9.4	10.6	4.8	9.5
H4-H5	4.3-5.5	6.5	3.7	6.0
H5-H6	n.d.	1.7	0.8	1.5

Coupling constants were determined by ^{1}H NMR experiments (exp.). n.d.: values not determine due to peak overlap.

2.3. Chemical synthesis

2.3.1. General Experimental Details

All reagents were of a commercial grade and were used as received unless stated otherwise. Dichloromethane (DCM), tetrahydrofuran (THF) and *N*,*N*-dimethylformamide (DMF) were stored over 4 Å molecular sieves, which were dried *in vacuo* before use. Triethylamine was dried over KOH and distilled before using. All reactions were performed under an argon atmosphere unless stated otherwise. Solvents used for flash column chromatography were of pro analysis quality. Reactions were monitored by analytical thin-layer chromatography (TLC) using Merck aluminum sheets pre-coated with silica gel 60 with detection by UV absorption (254 nm) and by spraying with a solution of $(NH_4)_6Mo_7O_{24}\cdot H_2O$ (25 g/L) and $(NH_4)_4Ce(SO_4)_4\cdot H_2O$ (10 g/L) in 10% sulfuric acid followed by charring at ~150 ˚C or by spraying with an aqueous solution of $KMnO_4$ (7%) and K_2CO_3 (2%) followed by charring at ~150 °C. Column chromatography was performed manually using either Baker or Screening Device silica gel 60 (0.04 - 0.063 mm) or a Biotage Isolera™ flash purification system using silica gel cartridges (Screening devices SiliaSep HP, particle size 15-40 μm, 60A) in the indicated solvents. ^{1}H NMR and ^{13}C NMR spectra were recorded on Bruker DMX-600 (600/150 MHz) and Bruker AV-400 (400/100 MHz) spectrometer in the given solvent. Chemical shifts are given in ppm relative to the chloroform residual solvent peak or tetramethylsilane (TMS) as internal standard. Coupling constants are given in Hz. All given ^{13}C spectra are proton decoupled. The following abbreviations are used to describe peak patterns when appropriate: s (singlet), d (doublet), t (triplet), qt (quintet), m (multiplet), br (broad), ar (aromatic), app (apparent). 2D NMR experiments (HSQC, COSY and NOESY) were carried out to assign protons and carbons of the new structures and assignation follows the general numbering shown in cyclohexene **9**. High-resolution mass spectra (HRMS) of intermediates were recorded with a LTQ Orbitrap (Thermo Finnigan) and final compounds were recorded with an apex-QE instrument (Bruker). Optical rotations were measured on a Anton Paar MCP automatic polarimeter (Sodium D-line, λ = 589 nm). LC/MS analysis was performed on an LCQ Advantage Max (Thermo Finnigan) ion-trap spectrometer (ESI+) coupled to a Surveyor HPLC system (Thermo Finnigan) equipped with a C18 column (Gemini, 4.6 mm x 50 mm, 3 μm particle size, Phenomenex) equipped with buffers A: H_2O, B: acetonitrile (MeCN) and C: 1% aqueous TFA, or an Agilent Technologies 1260 Infinity LCMS with a 6120 Quadrupole MS system equipped with buffers A: H_2O, B: acetonitrile (MeCN) and C: 100 mM NH_4OAc. For reversed-phase HPLC-MS purifications an Agilent Technologies 1200 series prepLCMS with a 6130 Qudropole MS system was used equipped with buffers A: 50 mM NH_4HCO_3 in H_2O and B: MeCN.

(*R*,*E*)-3-(but-2-enoyl)-4-isopropyloxazolidin-2-one (**4**)[25], (2*R*,3*S*)-2,3-bis(benzyloxy) pent-4-enal (**5**)[26], (1*R*,2*R*,5*S*,6*S*)-5,6-bis(benzyloxy)-2-(hydroxymethyl)cyclohex-3-en-1-ol (**14**)[25], (4a*R*,7*S*,8*R*,8a*R*)-2-phenyl-4a,7,8,8a-tetrahydro-4*H*-benzo[*d*][1,3]dioxine-7,8-diol (**15**)[27], ((((1*R*,2*R*,3*S*,6*R*)-6-((benzyloxy)methyl) cyclohex-4-ene-1,2,3-triyl)tris(oxy))tris(methylene)) tribenzene (**16**)[28,29], (1*S*,2*R*,5*S*,6*S*)-5,6-bis(benzyloxy)-2-(hydroxymethyl)cyclohex-3-en-1-ol (**17**)[30], (1*S*,4*S*,5*R*,6*R*)-4,5,6-tris(benzyloxy)cyclohex-2-en-1-ol (**19**)[31], ((((1*S*,2*R*,3*R*,4*S*)-cyclohex-5-ene-1,2,3,4-tetrayl)tetrakis(oxy))tetrakis(methylene))tetrabenzene (**20**)[27], (1*R*,5*S*,6*S*)-5,6-bis(benzyloxy)cyclohex-3-en-1-ol (**21**)[32], ((((1R,2S,3S)-cyclohex-4-ene-1,2,3-triyl)tris(oxy))tris(methylene))tribenzene (**22**)[32] and 3-amino-2-(trifluoromethyl)-2,3-dihydroquinazolin-4(1*H*)-one[33] were synthesized following procedures previously described and their spectroscopic data are in agreement with those previously reported.

2.3.2. Synthesis and Characterization Data of Compounds 1-3

(*R*)-3-((2*R*,3*R*,4*S*,5*S*)-4,5-bis(benzyloxy)-3-hydroxy-2-vinylhept-6-enoyl)-4-isopropyloxazolidin-2-one (**6**).

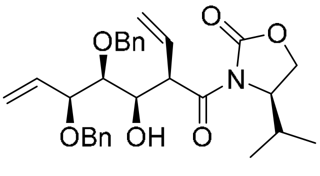

6

The reaction must be carried out under anhydrous conditions and argon atmosphere. Solutions of aldehyde and oxazolidinone in DCM were put under argon and dried with activated molecular sieves (4 Å) for at least 1 h prior to use. Et_3N was freshly distilled from CaH_2 and stored on activated molecular sieves (4 Å) under argon prior to use.

(4*R*)-3-[(2*E*)-but-2-enoyl]-4-isopropyl-1,3-oxazolidin-2-one (2.20 g, 11.2 mmol) was dissolved in anhydrous DCM (20 mL). Then, a solution of 1.0 M dibutylboryltrifluoromethanesulfonate (DBBT) in anhydrous DCM (11.2 mL, 11.2 mmol) was added at –78°C. The resulting dark yellow mixture was removed from the cold bath to dissolve any frozen triflate and cooled again back to –78°C. Et_3N (recently distilled, 1.79 mL, 12.8 mmol) was then added, causing the dark green color to fade. The solution was stirred for 1 h at –78°C and then at 0°C for 15 min and the solution turned yellow. While the reaction mixture was being cooled back down to –78°C, a solution of 2,3-di-*O*-benzyl-4,5-dideoxy-L-*threo*-pent-4-enose (3.31 g, 11.2 mmol) in anhydrous DCM (20 mL) was added to the reaction mixture via a cannula and argon atmosphere. The bath temperature was raised to –20 °C over 1 h and then the reaction mixture was stirred at this temperature overnight. The resulting yellow solution then warmed to –5°C and quenched with pH 7 phosphate buffer solution (25 mL). A 30% H_2O_2 solution (30 mL) was then added dropwise while maintaining the internal temperature below 5°C. Addition of the peroxide was continued until the internal temperature remained constant. The mixture was stirred for additional 30 min while slowly warming to room temperature. The reaction was then poured over aqueous saturated $NaHCO_3$ solution (100 mL) and the aqueous layer extracted with DCM (3 x 100 mL). The combined organic layers were dried over $MgSO_4$, filtered, and concentrated under reduced pressure. The crude product was purified by silica column chromatography (from pentane to pentane/EtOAc, 8:2) to afford the desired product **6** as colorless oil (3.32 g, 6.73 mmol, 60%). $[\alpha]_D^{20}$ = –2.4 (*c* = 1.0, $CHCl_3$). ^1H NMR (400 MHz, $CDCl_3$): *δ* = 7.39 – 7.25 (m, 10H, CH Ar), 6.00 – 5.84 (m, 2H, 2C*H*=CH_2), 5.47 – 5.24 (m, 4H, 2CH=C*H*$_2$), 4.89 (d, *J* = 10.9 Hz, 1H, CH*H* Bn), 4.75 (t, *J* = 8.6 Hz, 1H, C*H*-CH=CH_2), 4.64 (d, *J* = 11.6 Hz, 1H, CH*H* Bn), 4.61 (d, *J* = 10.9 Hz, 1H, CH*H* Bn), 4.42 (d, *J* = 11.6 Hz, 1H, CH*H* Bn), 4.24 – 4.15 (m, 3H, 3CH), 4.13 – 4.01 (m, 2H, CH_2 oxazolidinone), 3.51 (dd, *J* = 7.3, 1.8 Hz, 1H, C*H*), 2.34 – 2.23 (m, 2H, C*H*(CH_3)$_2$, OH), 0.81 (dd, *J* = 27.7, 7.0 Hz, 6 H, 2CH_3). ^{13}C NMR (101 MHz, $CDCl_3$): *δ* = 172.1, 153.1 (2C=O), 138.5, 138.2 (2C_q Ar), 135.1, 133.9 (2*C*H=CH_2), 128.8, 128.5, 128.4 127.9, (8CH Ar), 127.8, 127.6 (2CH Ar), 120.2, 120.1 (2CH=*C*H$_2$), 83.3 (CH), 80.1 (CH oxazolidinone), 74.6 (CH_2Bn), 71.4 (CH), 70.8 (CH_2Bn), 62.9 (CH_2 oxazolidinone), 58.3 (CH), 50.7 (*C*H-CH=CH_2), 28.1 [CH(CH_3)$_2$], 18.0, 14.50 (2CH_3) ppm. HRMS: calcd. for $[C_{29}H_{36}NO_6]^+$ 494.25371; found 494.25379. HRMS: calcd. for $[C_{29}H_{35}NO_6Na]^+$ 516.23566; found 516.23519.

3,4-di-*O*-benzyl-1,2,6-trideoxy-6-vinyl-L-*ido*-hept-1-enitol (**7**).

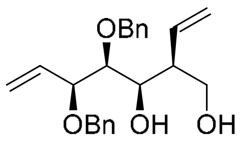

7

To a solution of oxazolidinone **6** (2.55 g, 5.2 mmol) in THF (50 mL) at 0°C, H_2O (2.5 mL) and $LiBH_4$ (2 M solution in THF, 12.9 mL, 25.8 mmol) were added and stirred 1 h at 0°C. The reaction mixture was then warmed to room temperature and stirred for 1 h. The reaction was quenched with 2 N NaOH (aq) (50 mL) and diluted with Et_2O (50 mL). After stirring for five minutes, the reaction mixture was poured over Et_2O (50 mL) and the separated organic phase was washed with aqueous saturated $NaHCO_3$ solution (20 mL) and brine (100 mL). The organic phase was dried over $MgSO_4$, filtered, and concentrated under reduced pressure to give the crude alcohol. The crude was purified by silica column chromatography (pentane/EtOAc from 9:1 to 1:1) to afford the desired product **7** as a colourless oil (1.89 g, 5.13 mmol, 99%). $[\alpha]_D^{20}$ = +6.0 (*c* = 0.5, $CHCl_3$). ^1H NMR (400 MHz, $CDCl_3$): *δ* = 7.38 – 7.27 (m, 10H, CH Ar), 5.98 – 5.81 (m, 2H, 2C*H*=CH2), 5.40–5.35 (m, 2 H, 2CH=CH*H*), 5.19 (dd, *J* = 10.3, 2.0 Hz, 1H, CH=CH*H*), 5.03 (dd, *J* = 17.3, 1.6 Hz, 1H, CH=CH*H*), 4.83 (d, *J* = 11.1 Hz, 1H,CH*H* Bn), 4.66 (d, *J* = 11.9 Hz, 1H, CH*H* Bn), 4.55 (d, *J* = 11.1 Hz, 1H, CH*H* Bn), 4.35 (d, *J* = 11.9 Hz, 1H, CH*H* Bn), 4.00 (dd, *J* = 7.6, 4.8 Hz, 1H, C*H*-OBn), 3.94 (dd, 8.9, 4.9 Hz, 1H, C*H*-OH), 3.62–3.60 (m, 2H, CH_2-OH), 3.43 (t, *J* = 5.1 Hz, 1 H, C*H*-OBn), 2.55 (br. s, 1 H, OH), 2.14 – 2.08 (m, 1H,C*H*-CH=CH_2), 2.02 (br. s, 1H, OH) ppm. ^{13}CNMR (101 MHz, $CDCl_3$): *δ* = 138.2, 138.10 (2C_q Ar), 135.4, 135.3 (2*C*H=CH_2), 128.6, 128.5, 128.4, 128.3 (8CH Ar), 128.1, 127.9 (2CH Ar), 119.4, 118.7 (2CH=*C*H_2), 82.3, 80.2 (2*C*H-OBn), 74.9 (CH_2 Bn), 71.3 (CH-OH), 70.6 (CH_2 Bn), 64.9 (CH_2-OH), 49.1 (*C*H-CH=CH_2) ppm. HRMS: calcd. for $[C_{23}H_{29}O_4]^+$ 369.20604; found 369.20725. HRMS: calcd. for $[C_{23}H_{28}NaO_4]^+$ 391.18798; found 391.18901.

(1*R*,2*S*,5*S*,6*S*)-5,6-bis(benzyloxy)-2-(hydroxymethyl)cyclohex-3-en-1-ol (**8**).

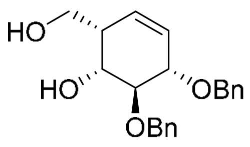

8

Second generation Grubbs catalyst (226 mg, 0.27 mmol) was added to a solution of **7** (1.96 g, 5.32 mmol, 1 equiv.) in DCM (200 mL), and the reaction mixture was stirred at 40°C protected from light for 24 h. Then, the solvent was evaporated under reduced pressure and the crude was purified by silica column chromatography (pentane/EtOAc from 9:1 to 1:1) to afford the desired product **8** as a dark brown oil (1.77 g, 5.20 mmol, 98%). $[\alpha]_D^{20}$ = +2.0 (*c* = 1, $CHCl_3$). ^1H NMR (400 MHz, $CDCl_3$): *δ* = 7.43 – 7.27 (m, 10H), 5.81 (ddd, *J* = 10.2, 2.5, 1.9 Hz, 1H, C*H*=CH-1), 5.66 (ddd, *J* = 10.2, 4.1, 1.5 Hz, 1H, C*H*=CH-6), 4.94 (d, *J* = 11.5 Hz, 1H, CH*H* Bn), 4.71 (d, *J* = 11.8 Hz, 1H, CH*H* Bn), 4.68 (d, *J* = 11.2 Hz, 1H, CH*H* Bn), 4.62 (d, *J* = 11.5 Hz, 1H, CH*H* Bn), 4.09 – 4.07 (m, 2H, CH-2, CH-4), 3.85 – 3.80 (m, 2H, CH-3, C*H*H-OH), 3.69 (dd, *J* = 11.4, 2.7 Hz, 1H, C*H*H-OH), 3.24 (br s, 2H, 2OH), 2.82 (br s, 1H, CH-5). ^{13}C NMR (101 MHz, $CDCl_3$): *δ* = 138.2, 137.9 (2C_q Ar), 128.7, 128.6 (4CH Ar), 128.1, 128.0 (2CH Ar), 128.0 (4CH Ar), 127.7, 127.0 (2*C*H=CH), 78.6, 78.5 (CH-3, CH-4/2), 74.2, 71.5 (2CH_2 Bn), 71.2 (CH-4/2), 63.9 (CH_2-OH), 41.9 (CH-5). HRMS: calcd. for $[C_{21}H_{25}O_4]^+$ 341.17528; found 341.17487. HRMS: calcd. for $[C_{21}H_{24}O_4Na]^+$ 363.15723; found 363.15662.

[({(1*R*,2*S*,5*S*,6*R*)-2,6-bis(benzyloxy)-5-[(benzyloxy)methyl]cyclohex-3-en-1-yl}oxy)methyl] benzene (**9**).

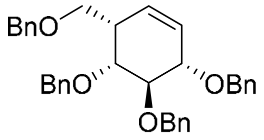

9

Catalytic amount of TBAI (109 mg, 0.29 mmol), BnBr (1.75 mL, 14.7 mmol) and sodium hydride (60% dispersion in mineral oil, 587 mg, 14.7 mmol) were added to a solution of **8** in DMF (120 mL) at 0 ºC and the reaction mixture was stirred at room temperature 24 h. The reaction mixture was cooled to 0 °C and quenched by the addition of MeOH. The mixture was further diluted with water and subsequently extracted with Et_2O. The organic phase was washed with brine, dried over $MgSO_4$, filtered, and concentrated in vacuo. The crude was purified by silica column chromatography (from pentane to pentane/EtOAc 9:1) to afford the desired product **9** (1.36 g, 89%) as colourless oil. $[\alpha]_D^{20}$ = –40 (*c* = 0.5, $CHCl_3$). ^{1}H NMR (400 MHz, $CDCl_3$): *δ =* 7.41 – 7.17 (m, 20H, 20CH Ar), 5.83 (ddd, *J* = 10.1, 4.3, 1.6 Hz, 1H, C*H*=CH-6), 5.75 (d, *J* = 10.2 Hz, 1H, C*H*=CH-1), 4.84 (d, *J* = 11.2 Hz, 1H, CH*H* Bn), 4.72 (d, *J* = 11.2 Hz, 1H, CH*H* Bn), 4.67 – 4.59 (m, 4H, 4CH*H* Bn), 4.52 (s, 2H, 2CH*H* Bn), 4.10 – 4.08 (m, 1 H, CH-4), 3.92 (dd, J = 9.2, 6.2 Hz, 1H, CH-3), 3.81 – 3.76 (m, 2H, CH-2, CH*H* Bn), 3.66 – 3.56 (t, *J* = 8.2 Hz, 1H, CH*H* Bn), 2.87 – 2.82 (m, 1H, CH-5). ^{13}C NMR (101 MHz, $CDCl_3$): *δ* = 139.0, 138.8, 138.8, 138.6 (4C_q Ar), 129.0 (*C*H=CH), 128.5 (4CH Ar), 128.5, 128.4, 128.1, 127.9, 127.8, 127.7(12CH Ar), 127.6 (3CH Ar), 127.6 (1CH Ar), 127.1 (*C*H=CH), 79.6 (CH-3), 79.4 (CH-4), 77.9 (CH-2), 74.6, 73.4, 72.5, 71.7 (4CH_2 Bn), 70.4 (2CH_2OBn), 39.6 (CH-5). HRMS: calcd. for $[C_{35}H_{37}O_4]^+$ 521.26918; found 521.26898. HRMS: calcd. for $[C_{35}H_{36}\,NaO_4]^+$ 543.25113; found 543.24976.

2-(trifluoromethyl)-3-{(1*R*,2*S*,3*S*,4*R*,5*S*,6*R*)-2,3,4-tris(benzyloxy)-5-[(benzyloxy)methyl]-7-azabicyclo[4.1.0]hept-7-yl}quinazolin-4(3*H*)-one (**10**).

A suspension of 2-trifluoromethyl-3-aminoquinazolin-4-one (2.55 g, 11.4 mmol) in anhydrous DCM (70 mL) was added dropwise over a period of 60 min to a stirred suspension of $PhI(OAc)_2$ (3.59 g, 11.1 mmol) in DCM (50 mL) at –25 ºC. The resultant mixture was stirred for additional 30 min and then a solution of the olefin **9** (2.9 g, 5.57 mmol) in DCM (20 mL) was added dropwise at a flowrate of 70 mL/h. The reaction mixture was then allowed to warm to oom temperature and stirred overnight. Then, the reaction mixture was diluted with brine (100 mL) and extracted with DCM (2 x 100 mL). The collected organic layers were dried over Na_2SO_4, filtered and the solvent was removed under reduced pressure and the crude was purified by silica column chromatography (from pentane to pentane/EtOaAC 8:2) to afford the desired aziridine **10** as a brown oil (1.79 g, 43%). $[\alpha]_D^{20}$ = + 1.2 (*c* = 1, $CHCl_3$).

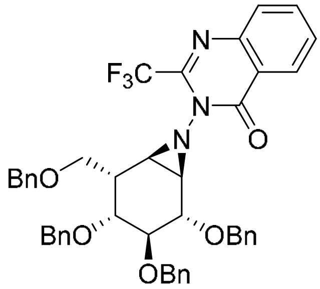

10

^{1}H NMR (400 MHz, $CDCl_3$): *δ* = 8.25 (d, *J* = 7.9 Hz, 1H, CH Ar), 7.87 (d, *J* = 3.6 Hz, 2H, 2CH Ar), 7.67 – 7.63 (m, 1H, CH Ar), 7.49 – 7.25 (m, 19H, 19CH Ar), 7.14 (t, *J* = 7.4 Hz, 1H, CH Ar), 4.87 (d, *J* = 11.1 Hz, 1H, CH*H* Bn), 4.84 (s, 2H, 2CH*H* Bn), 4.78 (d, *J* = 11.1 Hz, 1H, CH*H* Bn), 4.74 (d, *J* = 11.6 Hz, 1H, CH*H* Bn), 4.67 – 4.57 (m, 3H, 3CH*H* Bn), 4.22 (d, *J* = 7.3 Hz, 1H, CH-6), 4.06 (d, *J* = 7.0 Hz, 1H, CH-3), 4.00 (d, *J* = 7.4 Hz, 1H, CH-1), 3.98 – 3.96 (m, 1H, CH*H* Bn), 3.78 – 3.69 (m, 3H, CH-2, CH-4, CH*H* Bn), 2.99 – 2.93 (m, 1H, CH-5). ^{13}C NMR (101 MHz, $CDCl_3$): *δ* = 160.7 (C=O), 144.0 (C_q Ar Q), 143.4 (q, *J* = 34.1 Hz, CCF_3), 139.0, 138.6, 138.3, 138.1 (Cq Ar), 134.9, 129.3, 128.6, 128.5, 128.4, 128.0, 127.9, 127.8, 127.7, 127.6, 126.6 (24CH Ar), 123.2 (C Ar Q), 118.2 (q, *J* = 276.68 Hz, CF_3), 80.7 (CH-4/2), 80.2 (CH-3), 76.0 (CH-4/2), 75.2, 73.4, 73.2, 73.1 (4CH_2Bn), 67.6 (CH_2OBn), 44.2 (CH-6), 41.2 (CH-1), 38.0 (CH-5). HRMS: calcd. for $[C_{44}H_{40}F_3N_3O_5]^+$ 748.29983; found 748.29901. HRMS: calcd. for $[C_{44}H_{40}F_3N_3NaO_5]^+$ 770.28123; found 770.28076.

(1*R*,2*S*,3*S*,4*R*,5*S*,6*R*)-5-(hydroxymethyl)-7-azabicyclo[4.1.0]heptane-2,3,4-triol (**11**).

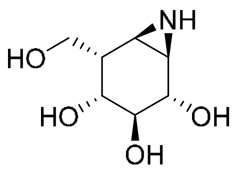

11

Ammonia (60 mL) was condensed at –60 °C. Lithium (0.67 g, 96 mmol) was added, and the mixture was stirred until the lithium was completely dissolved. A solution of aziridine **10** (1.79 g, 2.39 mmol) in THF (30 mL) was added. The reaction mixture was stirred for 1 h at –60 °C and subsequently quenched with milliQ-H_2O (20 mL). The solution was allowed to achieve room temperature and stirred until all ammonia was evaporated. Next, the solution was concentrated *in vacuo*, redissolved in milliQ-H_2O, filtered to remove the quinazolinone impurities and the filtrate was neutralized with Amberlite IR-120 H+. Product bound to the resin was washed with water and subsequently eluted with NH_4OH solution (1 M) and evaporated under reduced pressure to give the fully deprotected aziridine **11** as a beige foam (389 mg, 93%). ^{1}H NMR (400 MHz, D_2O): *δ* =2.47 (dd, *J* = 11.2, 4.4 Hz, 1H, CH*H*OH), 2.28 – 2.17 (m, 2H, CH*H*OH, CH-3), 2.11 (dd, *J* = 10.5, 5.6 Hz, 1H, CH-4), 1.98 (dd, *J* = 10.5, 7.4 Hz, 1H, CH-2), 1.07 (d, *J* = 6.0 Hz, 1H, CH-6), 1.01 (dt, *J* = 5.8, 5.1 Hz, 1H, CH-5), 0.77 (d, *J* = 6.0 Hz, 1H, CH-1). ^{13}C NMR (101 MHz, D_2O): *δ* = 76.0 (CH-2), 75.2 (CH-3), 70.1 (CH-4), 62.2 (CH_2), 43.5 (CH-5), 36.9 (CH-1), 36.3 (CH-6). HRMS: calcd. for $[C_7H_{14}NO_4]^+$ 176.09228; found 176.09175.

(1*R*,2*S*,3*S*,4*R*,5*S*,6*R*)-7-(8-azidooctyl)-5-(hydroxymethyl)-7-azabicyclo[4.1.0]heptane-2,3,4-triol (**12**).

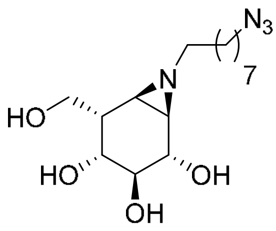

12

A solution of 1-azido-8-iodooctane (361 mg, 1.28 mmol) in 2 mL of anhydrous DMF was added to a solution of unprotected cyclophellitol aziridine **11** (150 mg, 0.86 mmol) and K_2CO_3 (533 mg, 3.85 mmol) in anhydrous DMF (5 mL). The reaction mixture was stirred at 55 °C for 18 h. Subsequently, the mixture was filtrated through a celite plug and concentrated under reduced pressure. Purification by silica column chromatography (from DCM to DCM/MeOH 9:1) yielded the desired product **12** (63 mg, 22%). $[\alpha]_D^{20}$ = –22.8 (*c* = 0.5, $CHCl_3$). ^{1}H NMR (400 MHz, CD_3OD): *δ* = 3.96 (dd, *J* = 10.8, 5.2 Hz, 1H, CH*H*OH), 3.75 (d, *J* = 6.3 Hz, 1H, CH-2), 3.68 – 3.61 (m, 2H, CH*H*OH, CH-4), 3.40 (dd, *J* = 8.8, 6.3 Hz, 1H, CH-3), 3.28 (t, *J* = 6.9 Hz, 3H, CH_2N_3), 2.42 – 2.37 (m, 1H, CH-5), 2.32 (t, *J* = 7.1 Hz, 2H, CH_2N), 1.92 (d, *J* = 6.1 Hz, 1H, CH-6), 1.72 (d, *J* = 6.1 Hz, 1H, CH-1), 1.59 (dd, *J* = 13.8, 6.9 Hz, 4H, $2CH_2$), 1.36 (s, 8H, $4CH_2$). ^{13}C NMR (101 MHz, CD_3OD): *δ* = 73.9 (CH-3), 72.6 (CH-2), 69.7 (CH-4), 61.8 (CH_2OH), 61.5 (CH_2N), 52.4 (CH_2N_3), 44.9 (CH-1), 42.8 (CH-6), 42.4 (CH-5), 30.5, 30.4, 30.2, 29.9, 28.3, 27.8 ($6CH_2$). HRMS: calcd. for $[C_{15}H_{29}N_4O_4]^+$ 329.21888; found 329.21820.

8-azido-1-((1*R*,2*S*,3*S*,4*R*,5*S*,6*R*)-2,3,4-trihydroxy-5-(hydroxymethyl)-7-azabicyclo[4.1.0] heptan-7-yl)octan-1-one (**13**).

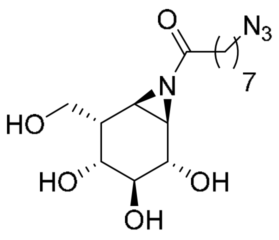

13

8-Azido-octanoic acid (170 mg, 0.92 mmol, 1.3 equiv.) and EEDQ (227 mg, 0.92 mmol, 1.3 equiv.) were dissolved in anhydrous DMF (1.0 mL) and stirred at room temperature for 2 h. This pre-activated mixed solution (500 µL, 0.65 equiv.) was added to a solution of aziridine **11** (124 mg, 0.71 mmol, 1.0 equiv.) in DMF (5 mL) at 0 °C and stirred for 30 minutes after which the remaining portion of the pre-activated solution (500 µL, 0.65 equiv.) was added. The resulting mixture was stirred at 0 °C for 3h. The reaction was quenched by 2 mL MeOH and the mixture was concentrated *in vacuo*. Then the crude product was purified by silica column chromatography (from DCM to DCM/MeOH 9:1) giving **13** as a colorless oil (56.3 mg, 0.16 mmol, 23%). $[\alpha]_D^{20}$ = –27.0 (*c* = 1, $CHCl_3$). ^{1}H NMR (400 MHz, CD_3OD): *δ* 3.92 (dd, *J* = 10.8, 4.5 Hz, 1H, C*H*HOH), 3.67 (d, *J* = 6.9 Hz, 1H, CH-2), 3.57 (dd, *J* = 10.8, 8.6 Hz, 1H, CH*H*OH), 3.52 (dd, *J* = 10.3, 5.4 Hz, 1H, CH-4), 3.32 (dd, *J* = 10.4, 6.8 Hz, 1H, CH-3), 3.19 (t, *J* = 6.9 Hz, 2H, CH_2N_3), 2.92 (dd, *J* = 5.8, 1.7 Hz, 1H, CH-6), 2.57 (d, *J* = 5.7 Hz, 1H, CH-1), 2.48 – 2.42 (m, 1H, CH-5), 2.36 (t, *J* = 7.4 Hz, 2H, CH_2CO), 1.59 – 1.44 (m, 4H, $2CH_2$), 1.33 – 1.22 (m, 6H, $3CH_2$). ^{13}C NMR (101 MHz, CD_3OD): *δ* 188.2 (C=O), 75.0 (CH-3), 73.5 (CH-2), 68.9 (CH-4), 60.6

(CH_2OH), 52.4 (CH_2N_3), 42.9 (CH-5), 42.2 (CH-1), 41.5 (CH-6), 37.2 (CH_2CO), 30.1, 29.9, 29.8, 27.6, 26.0 (5 CH_2). HRMS: calcd. for $[C_{15}H_{27}N_4O_5]^+$ 343.19814; found 343.19777.

(1*R*,2*R*,3*R*,4*S*,5*S*,6*R*)-7-(8-azidooctyl)-3,4,5-trihydroxy-7-azabicyclo[4.1.0]heptane-2-carboxylic acid (**1**).

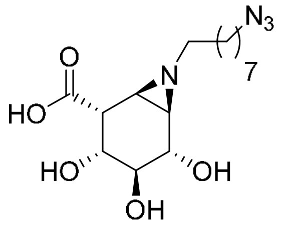

1

Intermediate **12** (25 mg, 0.076 mmol), catalytic amount of TEMPO (0.08 mg, 0.493 µmol), and NaBr (3.12 mg, 0.030 mmol) were dissolved in water (3.0 mL) at 0 °C. A 13% sodium hypochlorite solution (0.079 ml, 0.167 mmol) was added dropwise in 10 portions to the mixture, at the same time adjusted to pH=10.5 by adding an aqueous solution of NaOH (0.5 M). The reaction mixture was stirred at 0 ºC for 2 h. After which, starting material remained and more TEMPO (0.04 mg, 0.25 mmol), NaBr (1.6 mg, 0.015 mmol) was added and subsequent sodium hypochlorite (40 µL, 0.084 mmol) was added in 5 portions adjusting the pH to 10.5. After stirring 1 h at 0 ºC the oxidation was finished, the reaction was quenched by adding EtOH (1mL) and the pH was adjusted to 7 by adding an aqueous solution of HCl solution (0.5 M). The reaction mixture was lyophilized and purified by semi-preparative reversed HPLC (linear gradient: 19%→25% B in A, 12min, solutions used A: 50mM NH_4HCO_3 in H_2O, B: acetonitrile), the fractions were concentrated and lyophilized to afford desired product **1** as a white powder (3.6 mg, 14%). ^1H NMR (600 MHz, CD_3OD): δ = 3.67 (d, *J* = 6.8 Hz, 1H, CH-2), 3.55 (dd, *J* = 9.2, 5.5 Hz, 1H, CH-4), 3.46 (dd, *J* = 9.4, 6.8 Hz, 1H, CH-3), 3.28 (t, *J* = 6.9 Hz, 2H, CH_2N_3), 3.01 (d, *J* = 4.3 Hz, 1H, CH-5), 2.32 – 2.28 (m, 3H, CH_2N, CH-6), 1.66 (d, *J* = 6.1 Hz, 1H, CH-1), 1.62 – 1.53 (m, 4H, 2CH_2), 1.37 (d, *J* = 17.6 Hz, 8H, 4CH_2). ^{13}C NMR (151 MHz, CD_3OD): δ = 179.6 (COOH), 75.4 (CH-3), 73.4 (CH-2), 69.6 (CH-4), 61.7 (CH_2N), 52.5 (CH_2N_3), 46.5 (CH-5), 45.3 (CH-1), 43.8 (CH-6), 30.6, 30.5, 30.2, 29.9, 28.4, 27.8 (6CH_2). HRMS: calcd. for $[C_{15}H_{27}N_4O_5]^+$ 343.19814; found 343.19763.

(1*R*,2*R*,3*R*,4*S*,5*S*,6*R*)-7-(8-azidooctanoyl)-3,4,5-trihydroxy-7-azabicyclo[4.1.0]heptane-2-carboxylic acid (**14**).

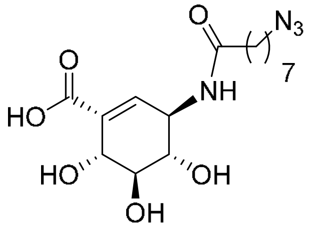

Intermediate **13** (23 mg, 0.067 mmol, 1eq.), TEMPO (0.1 mg, 0.44 umol, 0.0065eq.), and NaBr (2.8 mg, 0.03 mmol, 0.4 eq.) were dissolved in water (5.0 mL) at 0 °C. A 13% sodium hypochlorite solution (0.07 ml, 0.148 mmol, 2.2eq.) was added dropwise in 10 portions to the mixture, while at the same time the pH was adjusted to pH=10.5 by adding aqueous 0.5 M NaOH. The reaction was conducted at 0 ºC in an ice bath and checked by LC/MS within the elution system of 10% NH4OAc. After 3 h product with the expected mass was detected and the reaction was quenched by adding 96% EtOH (1mL) and the pH was adjusted to 7 by adding aqueous 0.5 M HCl. The reaction mixture was freeze-dried and the crude product was purified by semi-preparative reversed HPLC (linear gradient: 14%→19% B in A, 12min, solutions used A: 50mM NH_4HCO_3 in H_2O, B: acetonitrile), the fractions were concentrated and lyophilized to white powder product (2 mg, 5.6 µmol, 8.4%). ^1H NMR (500 MHz, CD_3OD): δ 6.24 (t, *J* = 1.9 Hz, 1H, CH=C), 4.55 (dt, *J* = 9.0, 2.7 Hz, 1H, CH-1), 4.35 (ddd, *J* = 7.7, 3.1, 1.6 Hz, 1H, CH-2), 3.57 (dd, *J* = 10.2, 7.8 Hz, 1H, CH-3), 3.45 (dd, *J* = 10.2, 9.0 Hz, 1H, CH-4), 3.30 – 3.23 (m, 2H, CH_2), 2.23 (d, *J* = 7.6 Hz, 2H, CH_2), 2.15 (d, *J* = 7.6 Hz, 2H, CH_2), 1.62 – 1.55 (m, 4H, 2CH_2), 1.40 – 1.34 (m, 6H, CH_2).

General Click Procedure for ABPs 2 and 3.

A solution of $CuSO_4{\cdot}5H_2O$ and sodium ascorbate (0.1M in MilliQ water) was prepared and degassed before using. Azido cyclophellitol aziridine **1** (1 equiv.) was dissolved in DMF (2 mL) and $CuSO_4{\cdot}5H_2O$ (1 M, 0.45 equiv.) and sodium ascorbate (1 M, 0.48 equiv.) were added to the solution under argon atmosphere. Then, the corresponding alkyne (1 equiv.) was added and the reaction mixture was stirred at room temperature overnight. Then, the reaction mixture was concentrated under reduced pressure and purified by semi-preparative reversed HPLC (linear gradient: %→% B in A, 12min, solutions used A: 50mM NH_4HCO_3 in H_2O, B: acetonitrile), the fractions were concentrated and lyophilized to afford the desired product.

2-{(1*E*,3*E*,5*E*)-5-[1-(6-{[(1-{8-[(1*R*,2*R*,3*R*,4*S*,5*S*,6*R*)-2-carboxy-3,4,5-trihydroxy-7-azabicyclo[4.1.0]hept-7-yl]octyl}-1*H*-1,2,3-triazol-4-yl)methyl]amino}-6-oxohexyl)-3,3-dimethyl-1,3-dihydro-2*H*-indol-2-ylidene]penta-1,3-dien-1-yl}-1,3,3-trimethyl-3*H*-indolium chloride (**2**).

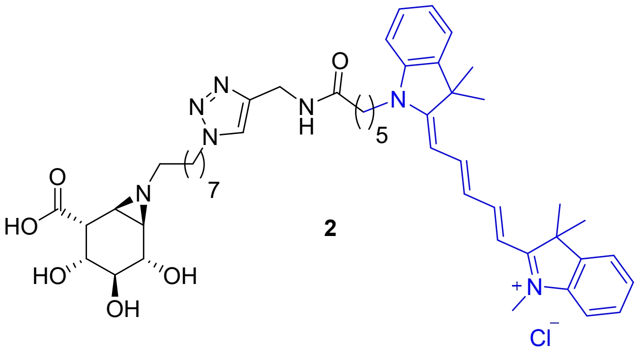

Obtained from azido **1** (5.3 mg, 15 µmol) and Cy5-alkyne (10.76 mg, 19 µmol) as a blue powder in 22% yield. Semi-preparative reversed HPLC (linear gradient: 41%→44% B in A, 12min, solutions used A: 50mM NH_4HCO_3 in H_2O, B: acetonitrile). ^{1}H NMR (600 MHz, CD_3OD): δ = 8.25 (t, *J* = 13.0 Hz, 2H, 2C*H*=CH), 7.85 (d, *J* = 5.5 Hz, 1H, CHN-N=N), 7.49 (d, *J* = 7.4 Hz, 2H, 2CH Ar), 7.43-7.40 (m, 2H, 2CH Ar), 7.31 – 7.25 (m, 4H, 4CH Ar), 6.62 (t, *J* = 12.4 Hz, 1H, C*H*=CH), 6.28 (d, *J* = 13.7 Hz, 2H, 2C*H*=CH), 4.41 (d, *J* = 4.6 Hz, 2H, CH_2), 4.37 (t, *J* = 7.0 Hz, 2H, CH_2), 4.09 (t, *J* = 7.4 Hz, 2H, CH_2), 3.66 – 3.65 (m, 1H, CH), 3.63 (s, 3H, CH_3), 3.56 – 3.54 (m, 1H, CH), 3.46 (dd, *J* = 9.0, 7.0 Hz, 1H, CH), 3.00 (d, *J* = 4.2 Hz, 1H, CH), 2.27 – 2.24 (m, 3H, CH, CH_2), 1.88 – 1.86 (m, 2H, CH_2), 1.83 – 1.79 (m, 2H, CH_2), 1.72 (s, 12H, 4CH_3), 1.71 – 1.68 (m, 2H, CH_2), 1.65 – 1.59 (m, 1H, CH), 1.53 – 1.50 (m, 2H, CH_2), 1.49 – 1.44 (m, 2H, CH_2), 1.40 – 1.38 (m, 2H, CH_2), 1.34 – 1.29 (m, 8H, 4CH_2). ^{13}C NMR (151 MHz, CD_3OD): δ = 175.7, 175.4, 174.6 (3C), 155.6, 155.5 (2*C*H=CH), 144.3, 143.6, 142.6, 142.5 (4C), 129.8, 129.7 (2CH Ar), 126.7 (*C*H=CH), 126.3, 126.2 (2CH Ar), 124.2 (CHN-N=N), 123.4, 123.3, 112.0, 111.9 (4CH Ar), 104.5, 104.3 (2*C*H=CH), 75.4, 73.5, 69.6, 47.3, 45.2 (5CH), 44.8 (CH_2), 43.8 (CH), 36.5, 35.6 (2CH_2), 31.5 (CH_3), 31.3, 31.2, 30.4, 30.4, 29.9, 28.2, 28.1 (7CH_2), 27.9, 27.8 (4CH_3), 27.3, 27.2, 26.4 (3CH_2). HRMS: calcd. for $[C_{50}H_{68}N_7O_6+]^+$ 862.52256; found 862.52215.

(1*R*,2*R*,3*R*,4*S*,5*S*,6*R*)-3,4,5-trihydroxy-7-{8-[4-({[6-({5-[(3a*S*,4*S*,6a*R*)-2-oxohexahydro-1*H*-thieno[3,4-*d*]imidazol-4-yl]pentanoyl}amino)hexanoyl]amino}methyl)-1*H*-1,2,3-triazol-1-yl]octyl}-7-azabicyclo[4.1.0]heptane-2-carboxylic acid (**3**).

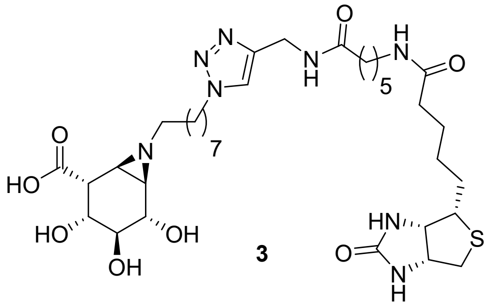

Obtained from azido **1** (3.0 mg, 8.8 µmol) and Biotyn-alkyne (4.3 mg, 11 µmol) as a white powder in 34% yield. Semi-preparative reversed HPLC (linear gradient: 16%→19% B in A, 12min, solutions used A: 50mM NH_4HCO_3 in H_2O, B: acetonitrile). ^{1}H NMR (600 MHz, CD_3OD): δ = 7.84 (d, *J* = 6.2 Hz, 1H, CHN-N=N), 4.58 (br s, 2H, 2NH), 4.49 (dd, *J* = 7.8, 5.0 Hz, 1H, C*H*NH), 4.42 (d, *J* = 2.9 Hz, 2H, CH_2), 4.38 (td, *J* = 7.0, 3.6 Hz, 2H, CH_2), 4.30 (dd, *J* = 7.8, 4.5 Hz, 1H, C*H*NH), 3.68 (dd, *J* = 10.7, 5.0 Hz, 1H, CH), 3.49 – 3.47 (m, 1H, CH), 3.35 – 3.30 (m, 3H, CH_2, CH), 3.20 (dd, *J* = 8.9, 4.4 Hz, 1H, CH), 3.17 – 3.12 (m, 3H), 2.93 (dd, *J* = 12.7, 5.0 Hz, 1H, C*H*HS), 2.70 (d, *J* = 12.7 Hz, 1H, C*H*HS), 2.31 – 2.29 (m, 1H, CH), 2.23 (t, *J* = 7.5 Hz, 2H, CH_2), 2.19 (t, *J* = 7.4 Hz, 2H, CH_2), 1.74 – 1.60 (m, 9H, 4CH_2, CH), 1.53 – 1.48 (m, 2H, CH_2), 1.46 – 1.41 (m, 2H, CH_2), 1.39 – 1.29 (m, 12H, 6CH_2). ^{13}C NMR (151 MHz, CD_3OD): δ = 179.7 (COOH), 176.0 (2CONH), 176.0, 166.1 (2NHCONH), 146.2 (C), 124.1 (CHN-N=N), 77.3, 72.3, 70.8 (3CH), 64.6 (CHS), 63.4, 61.6 (CHNH), 57.0 (CH), 51.4, 51.3 (2CH_2), 49.6, 45.2 (2CH), 41.1 (CH_2S), 40.2, 36.8, 36.7, 35.6, 31.3, 31.2, 30.5, 30.4, 30.1, 29.8, 29.5, 28.3, 27.5, 27.5, 27.4, 26.9, 26.5 (17CH_2). HRMS: calcd. for $[C_{34}H_{57}N_8O_8S]^+$ 737.40201; found 737.40167.

3. NMR spectra:

^{1}H-NMR and ^{13}C-NMR spectra of **6** in $CDCl_3$

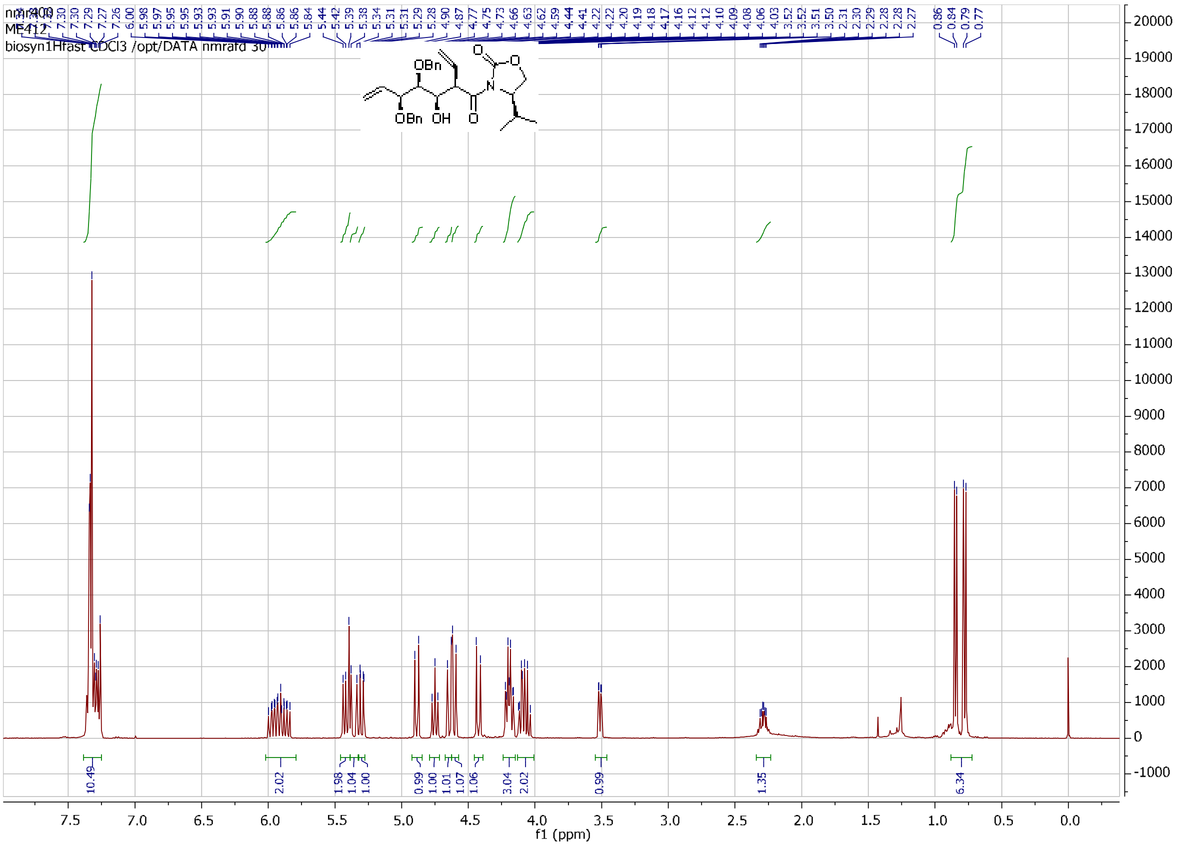

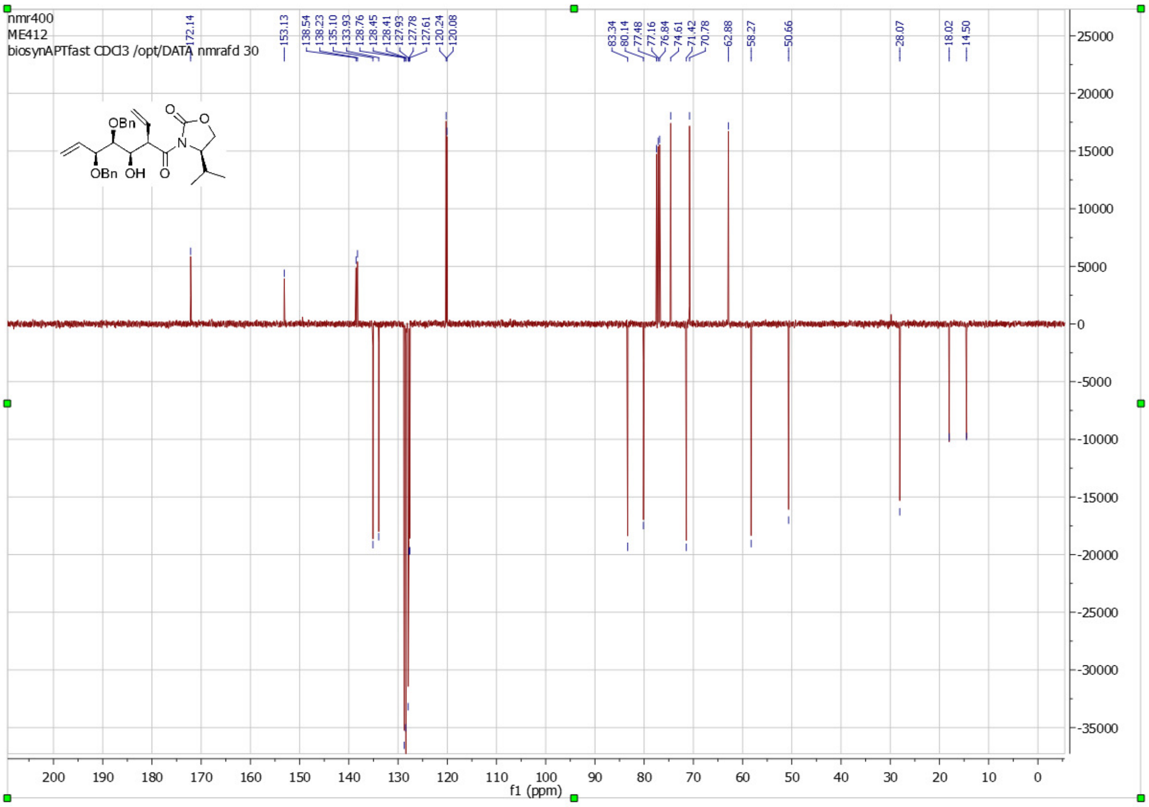

^{1}H-NMR and ^{13}C-NMR spectra of **7** in $CDCl_3$

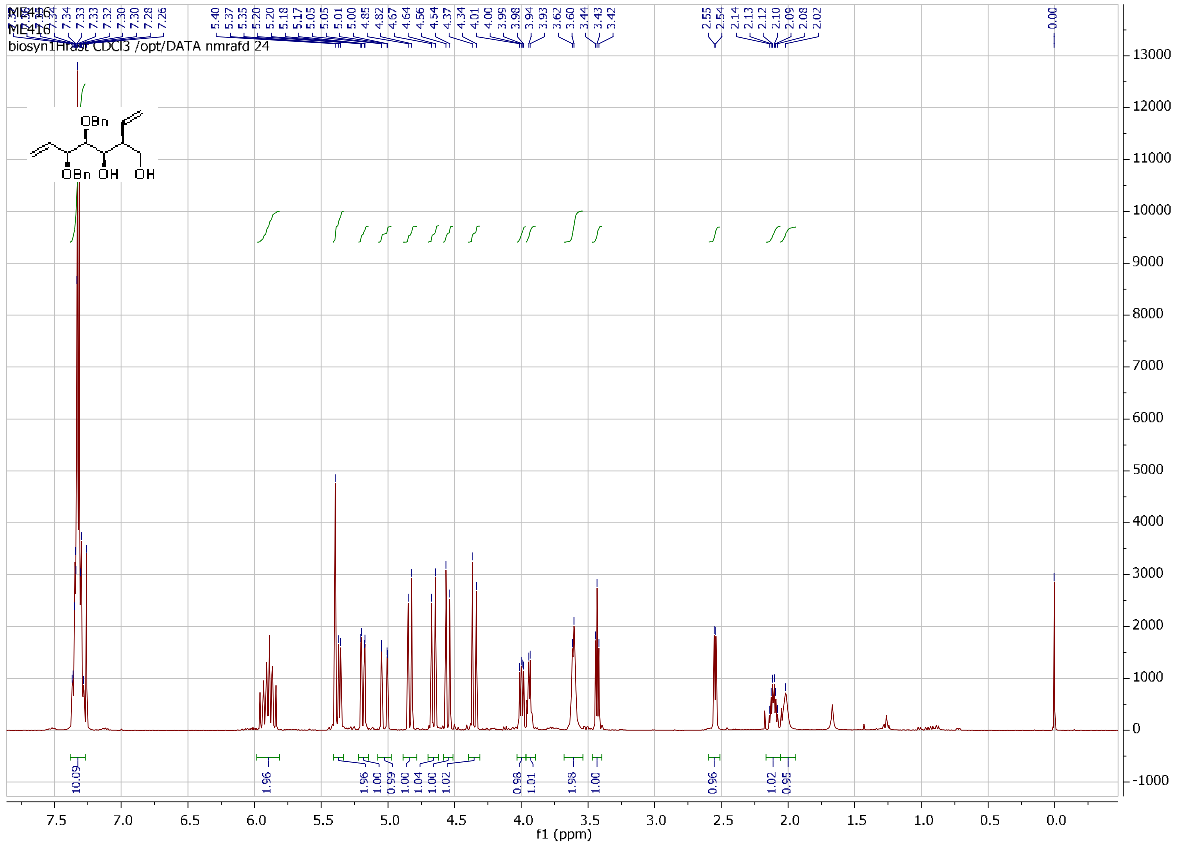

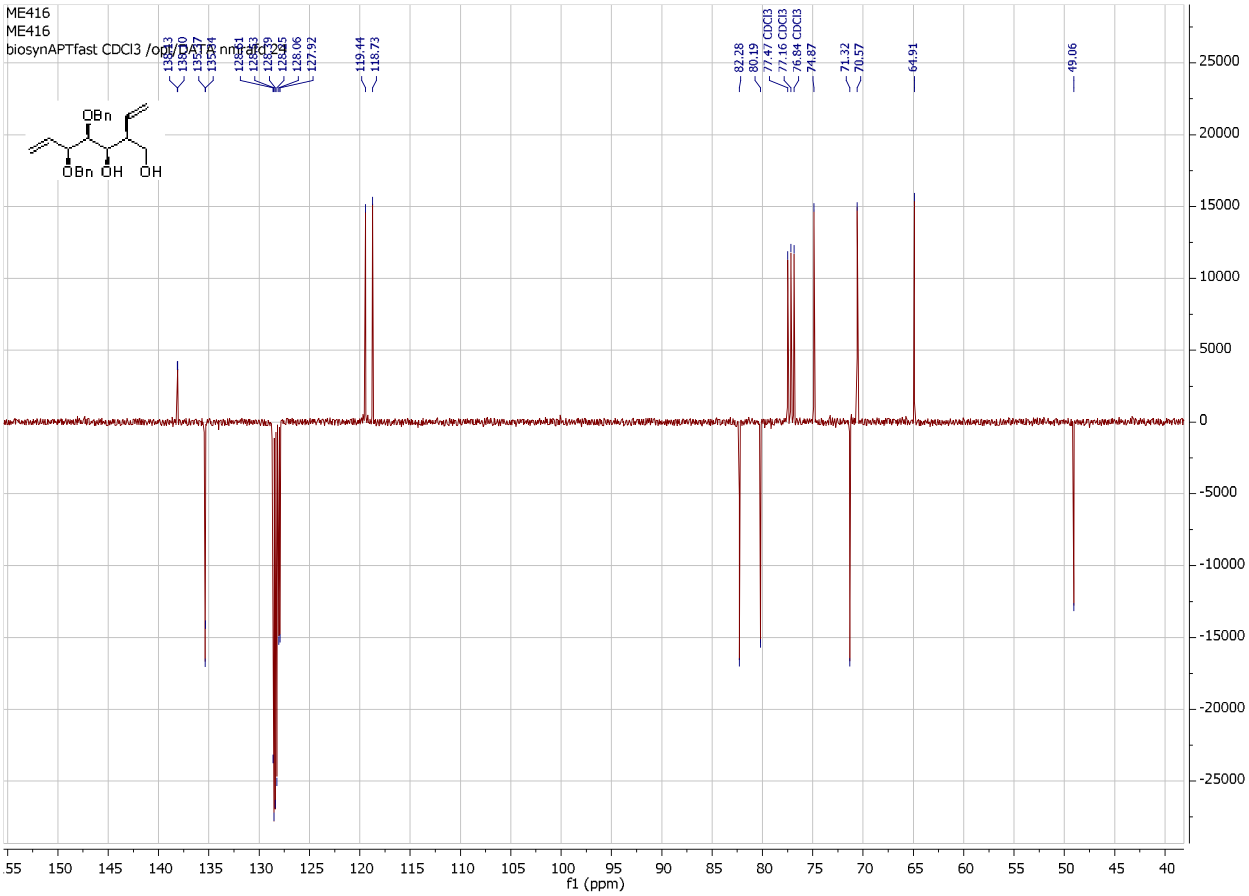

^{1}H-NMR and ^{13}C-NMR spectra of **8** in $CDCl_3$

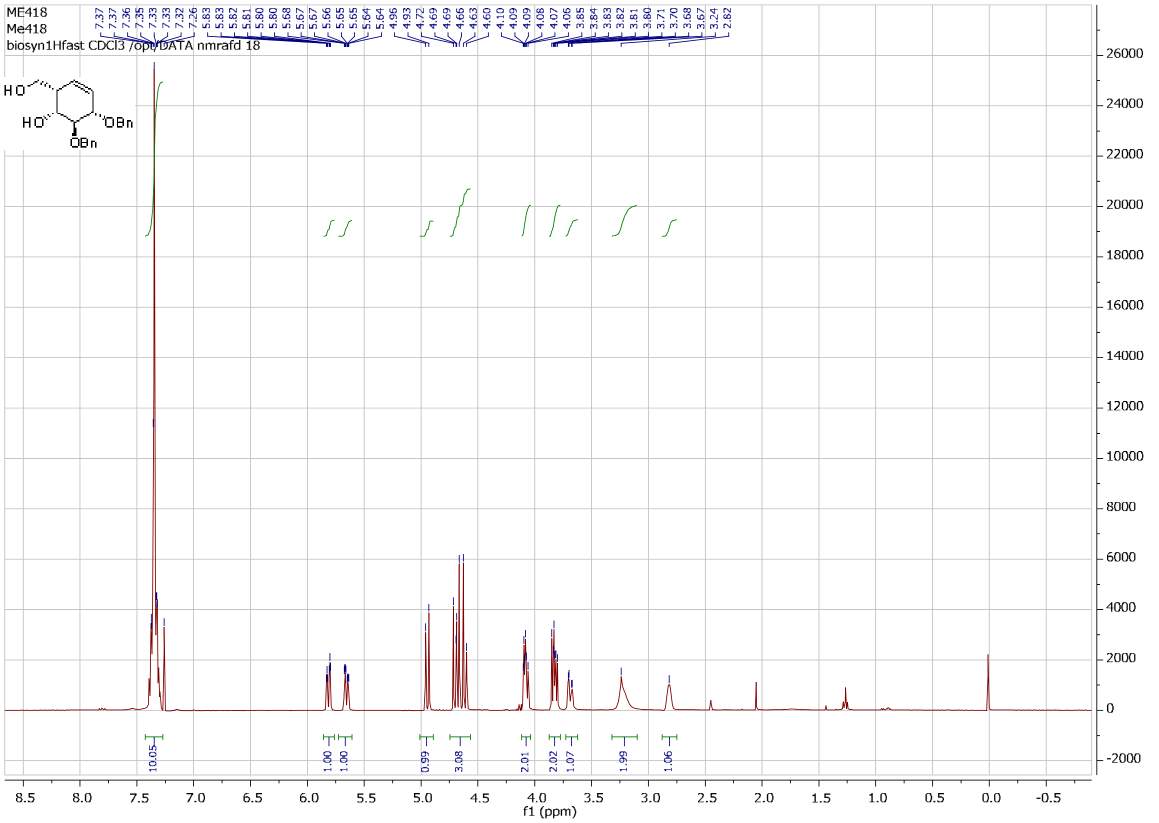

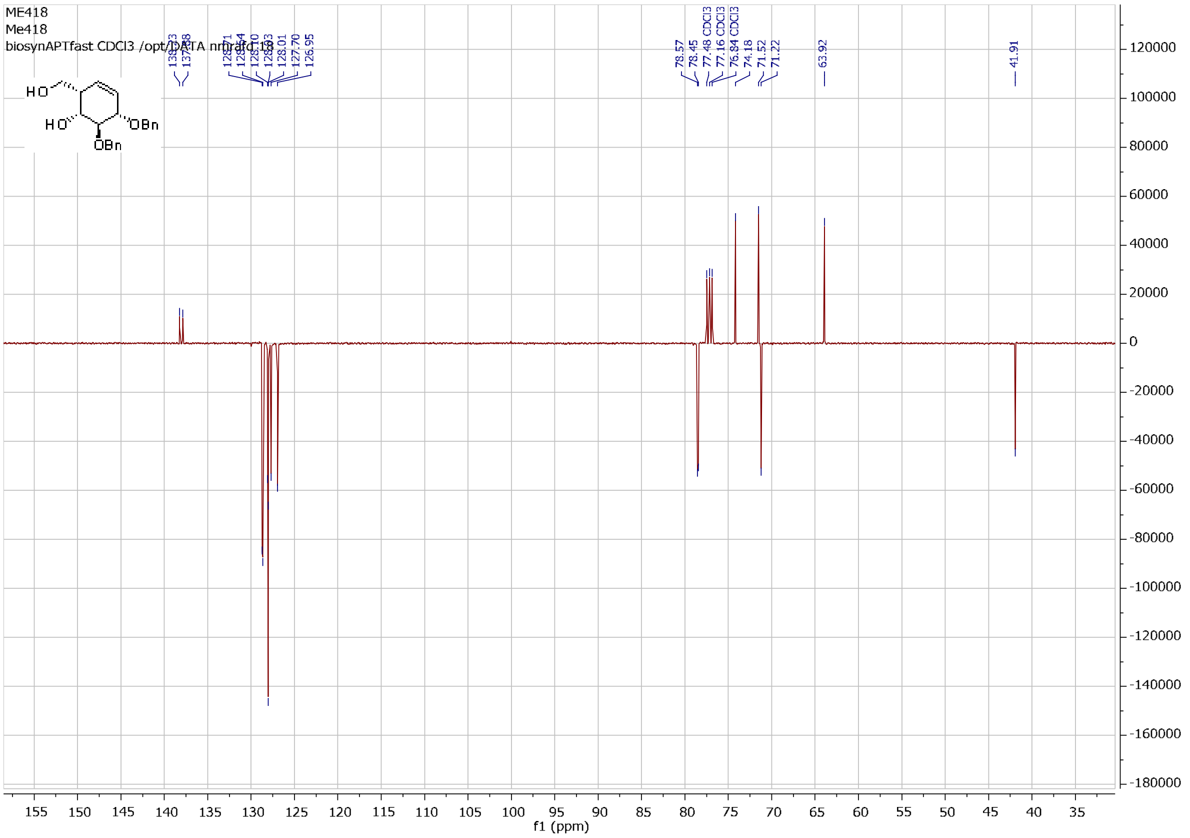

^{1}H-NMR and ^{13}C-NMR spectra of **9** in $CDCl_3$

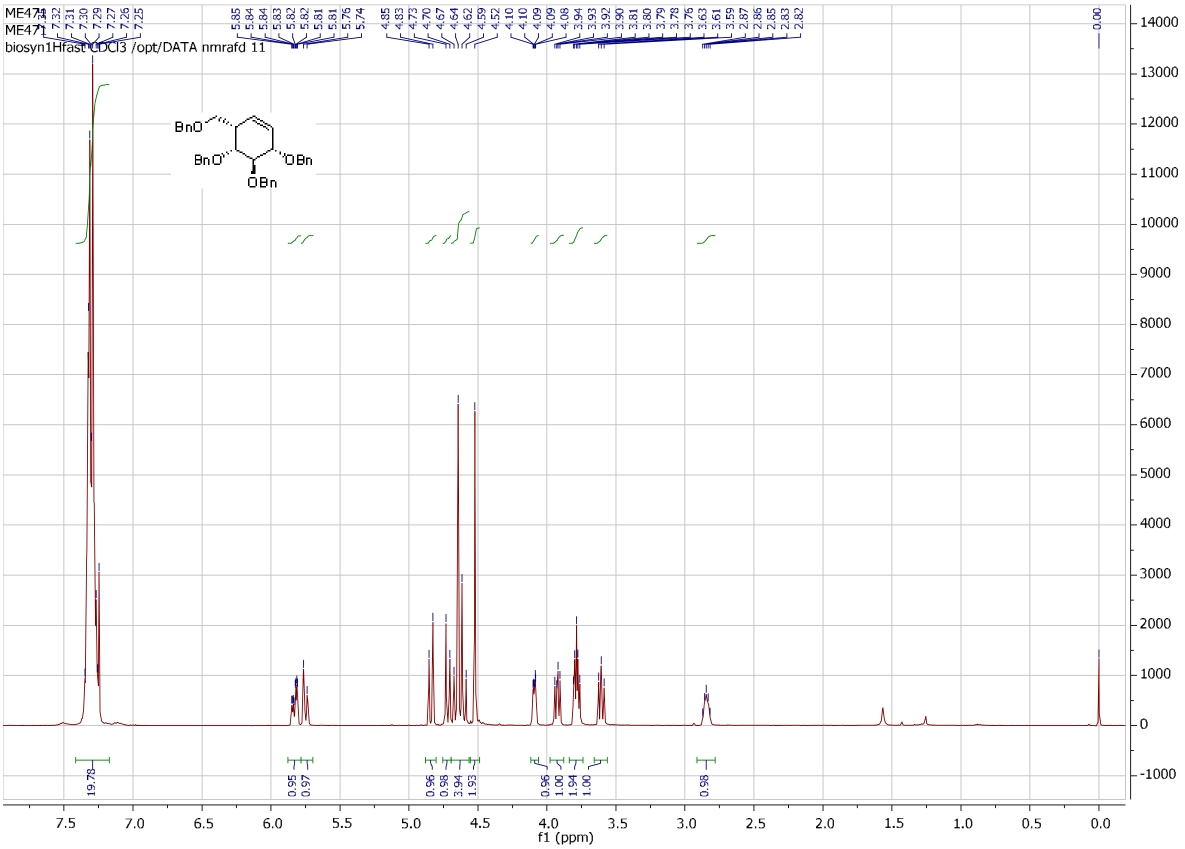

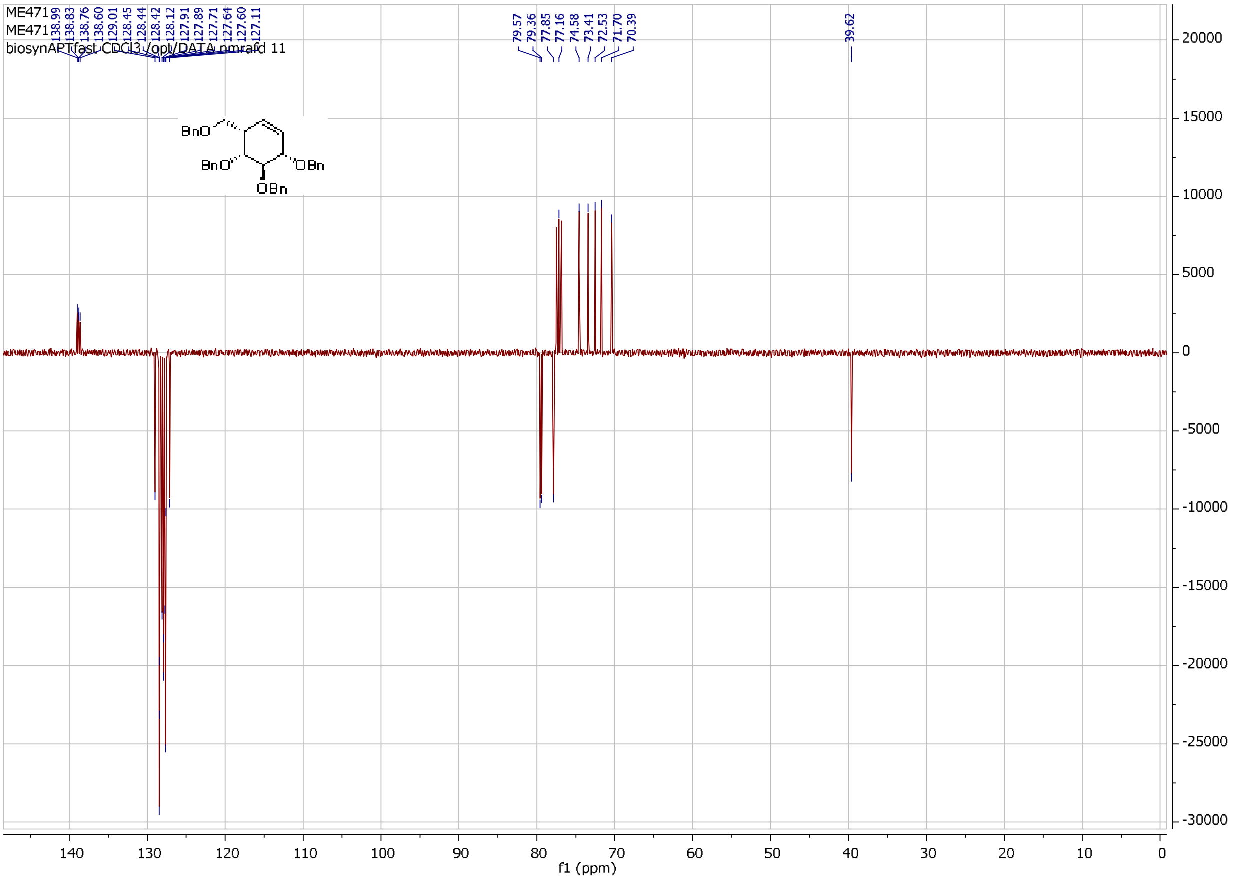

^{1}H-NMR and ^{13}C-NMR spectra of **10** in $CDCl_3$

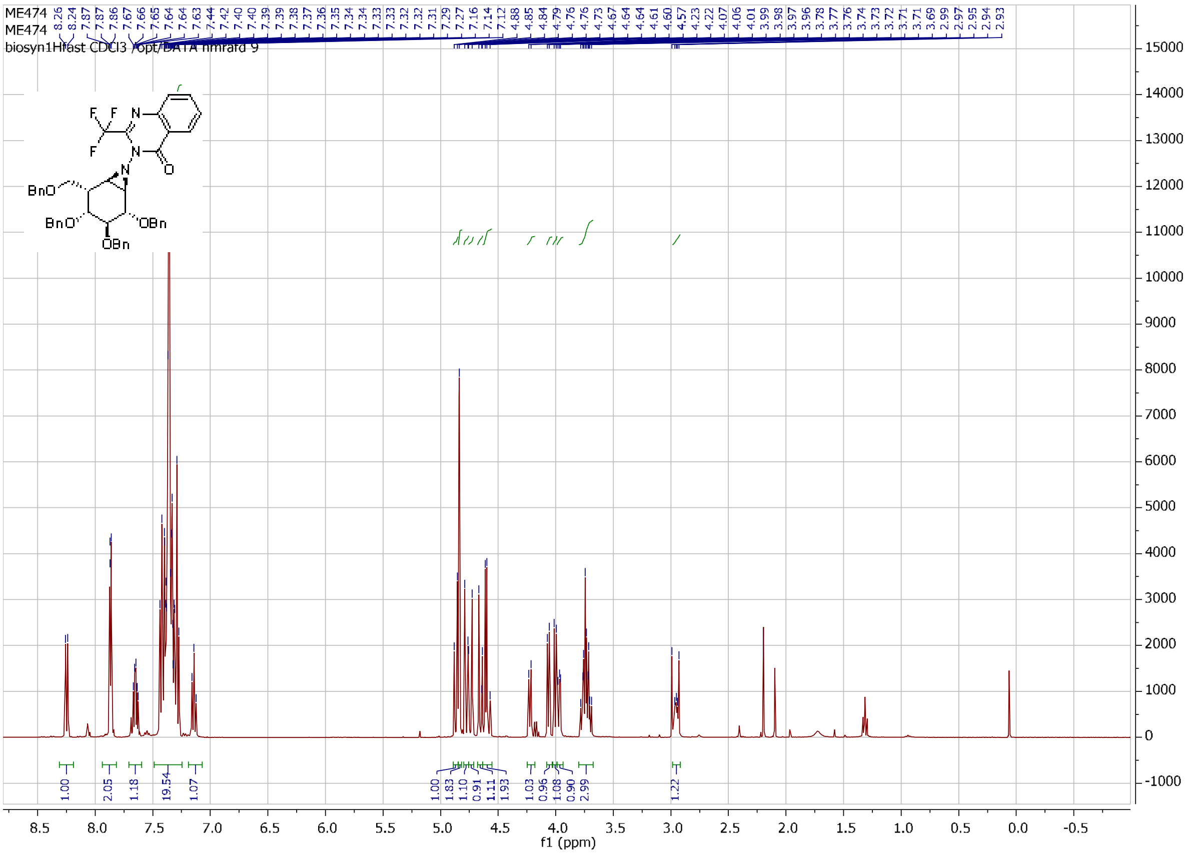

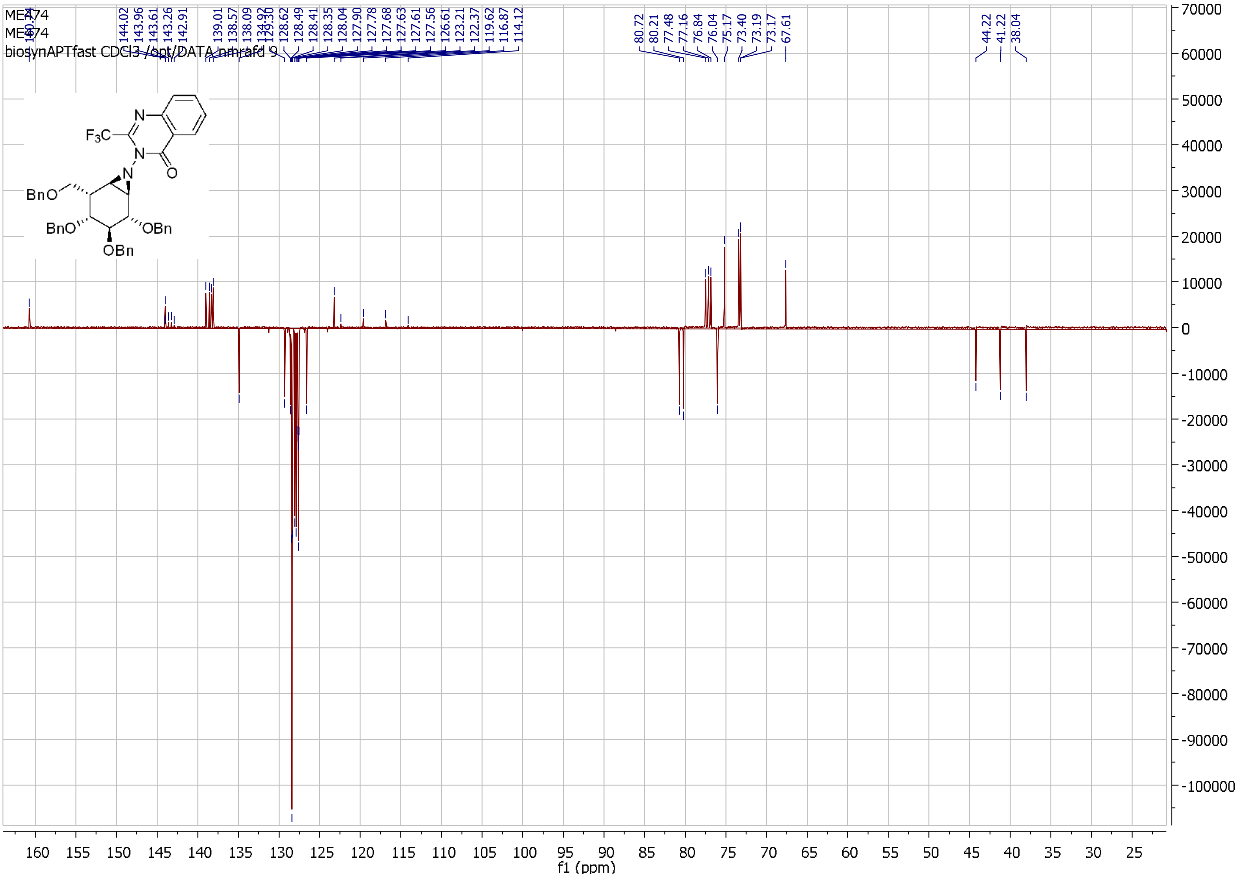

^{1}H-NMR and ^{13}C-NMR spectra of **11** in D_2O

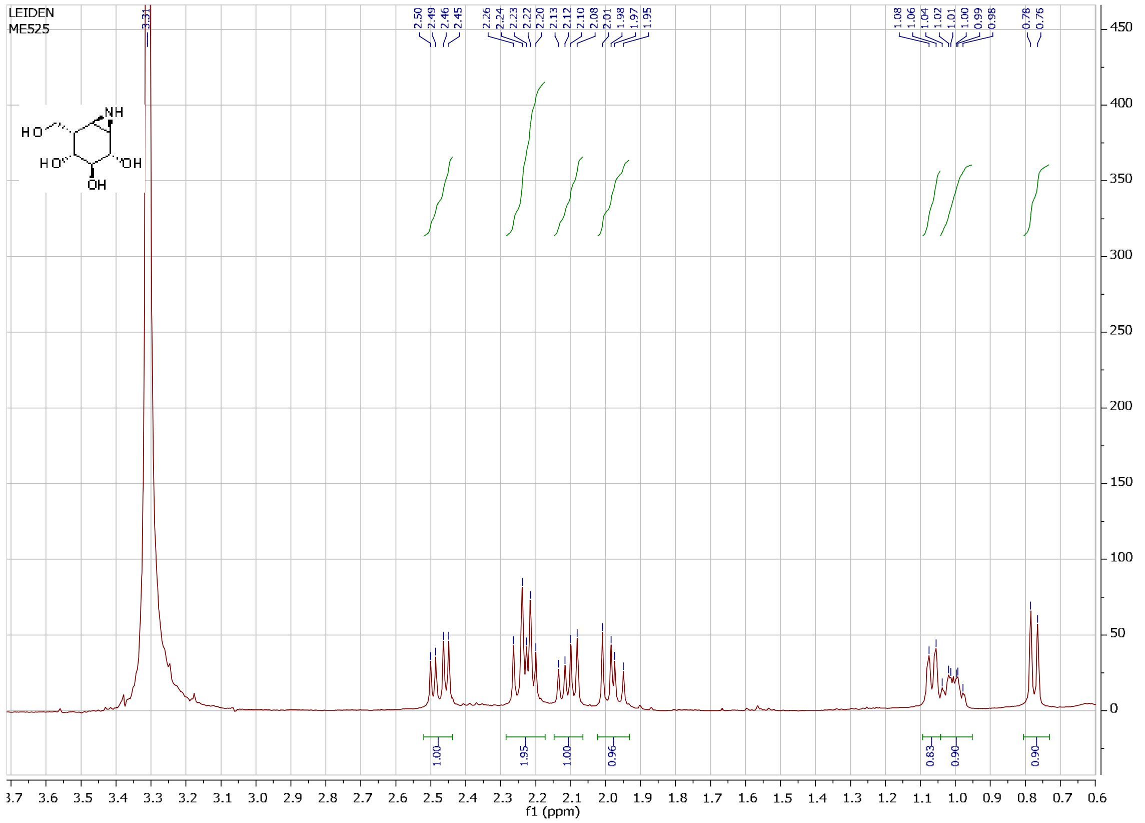

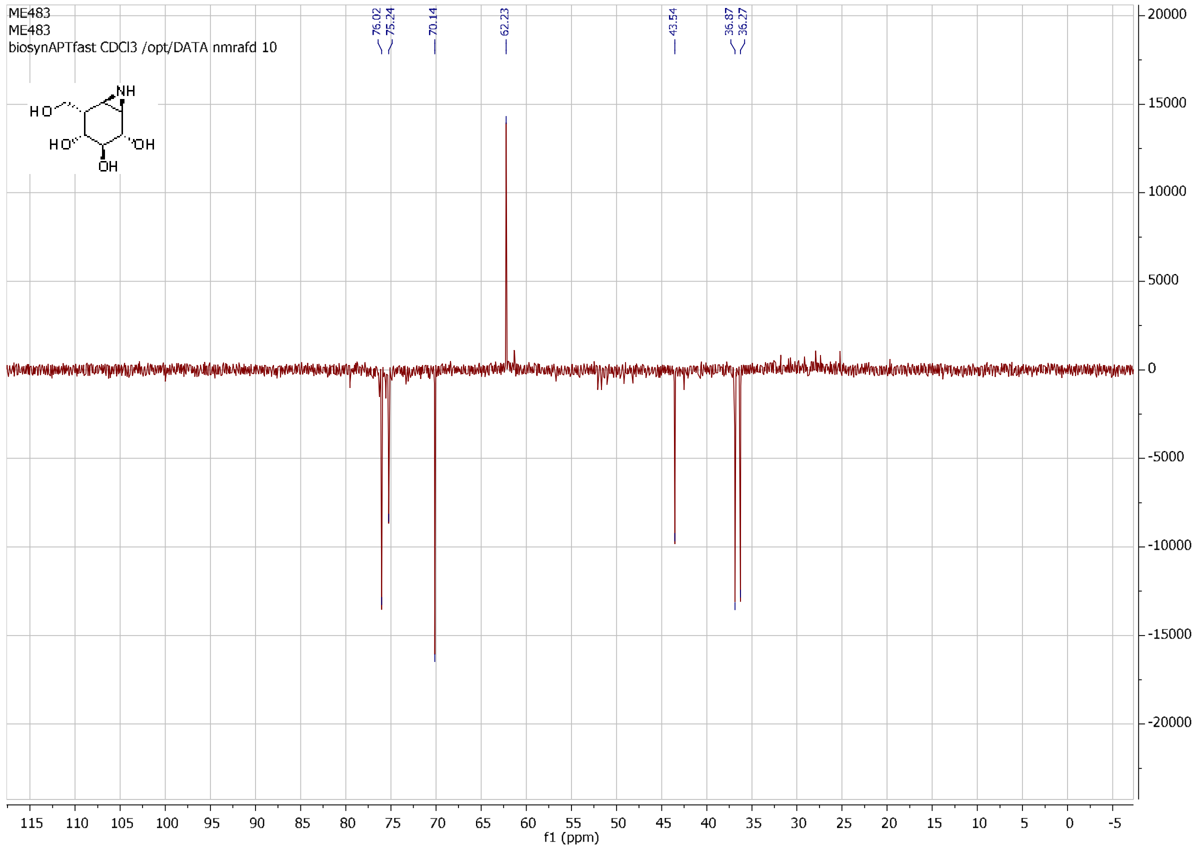

^{1}H-NMR and ^{13}C-NMR spectra of **12** in MeOD

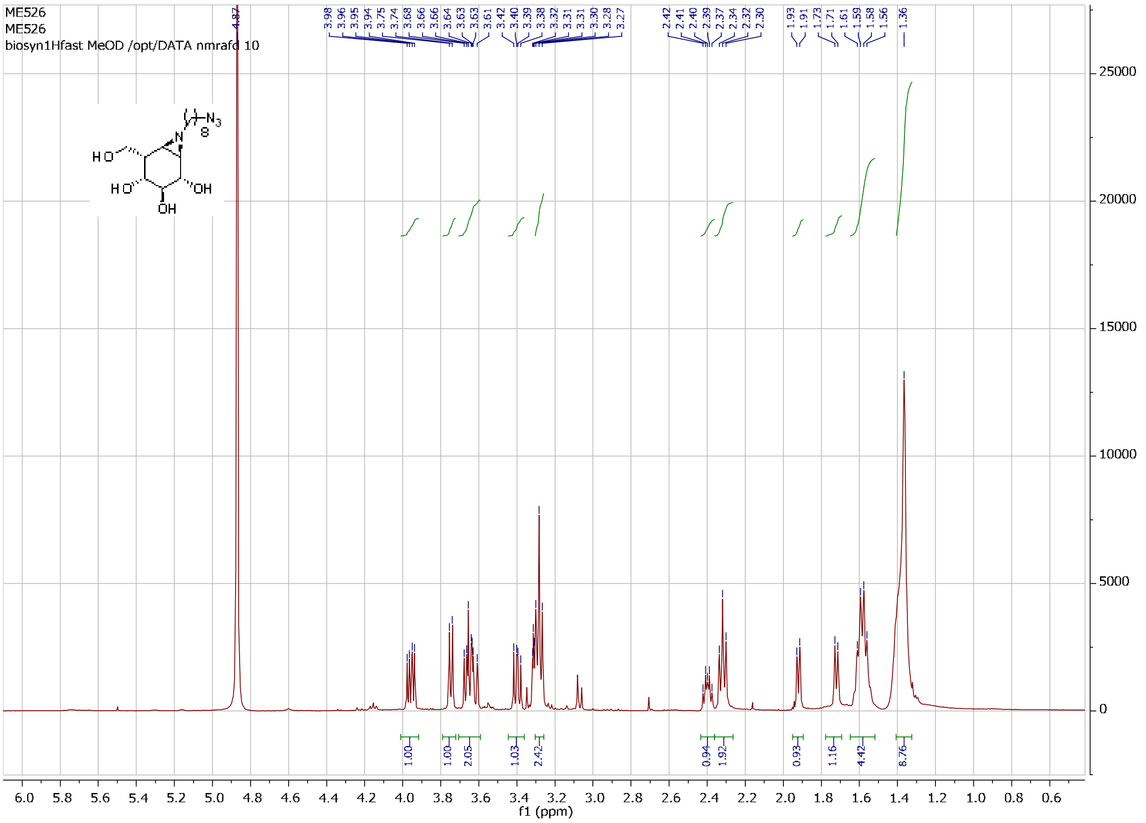

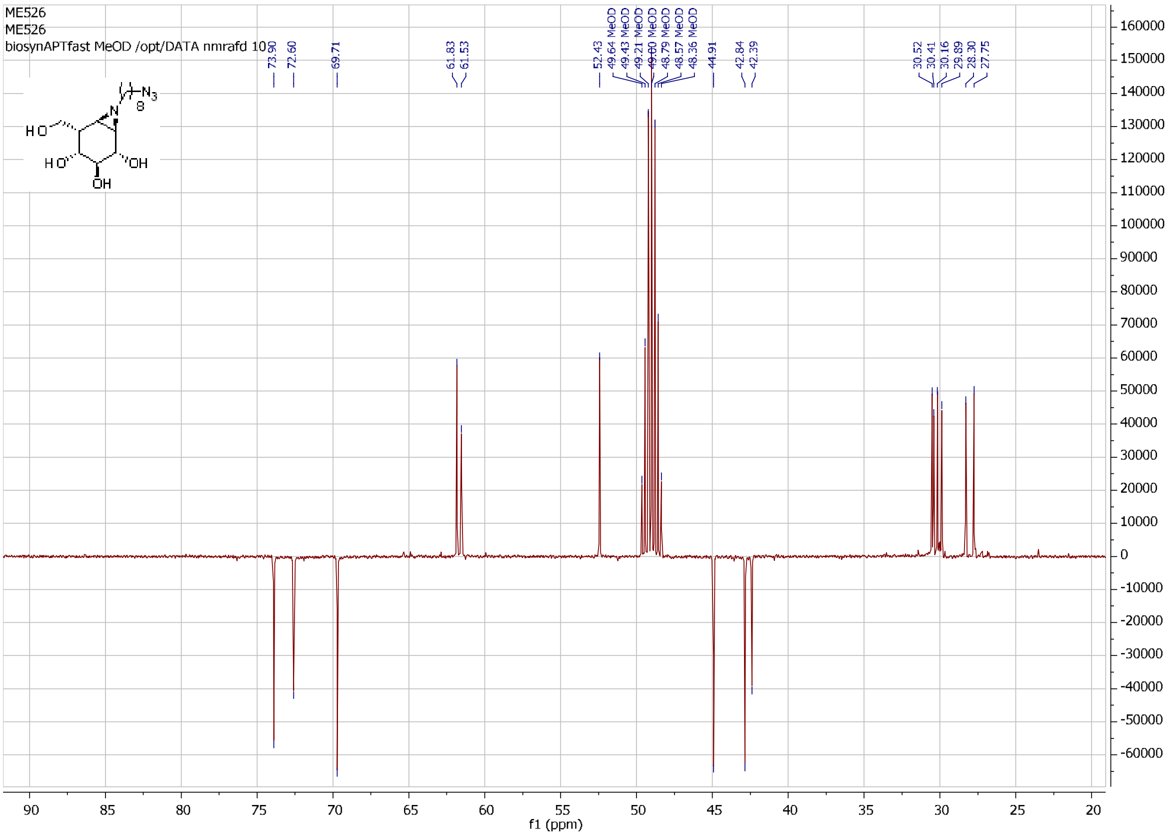

^{1}H-NMR and ^{13}C-NMR spectra of **1** in MeOD

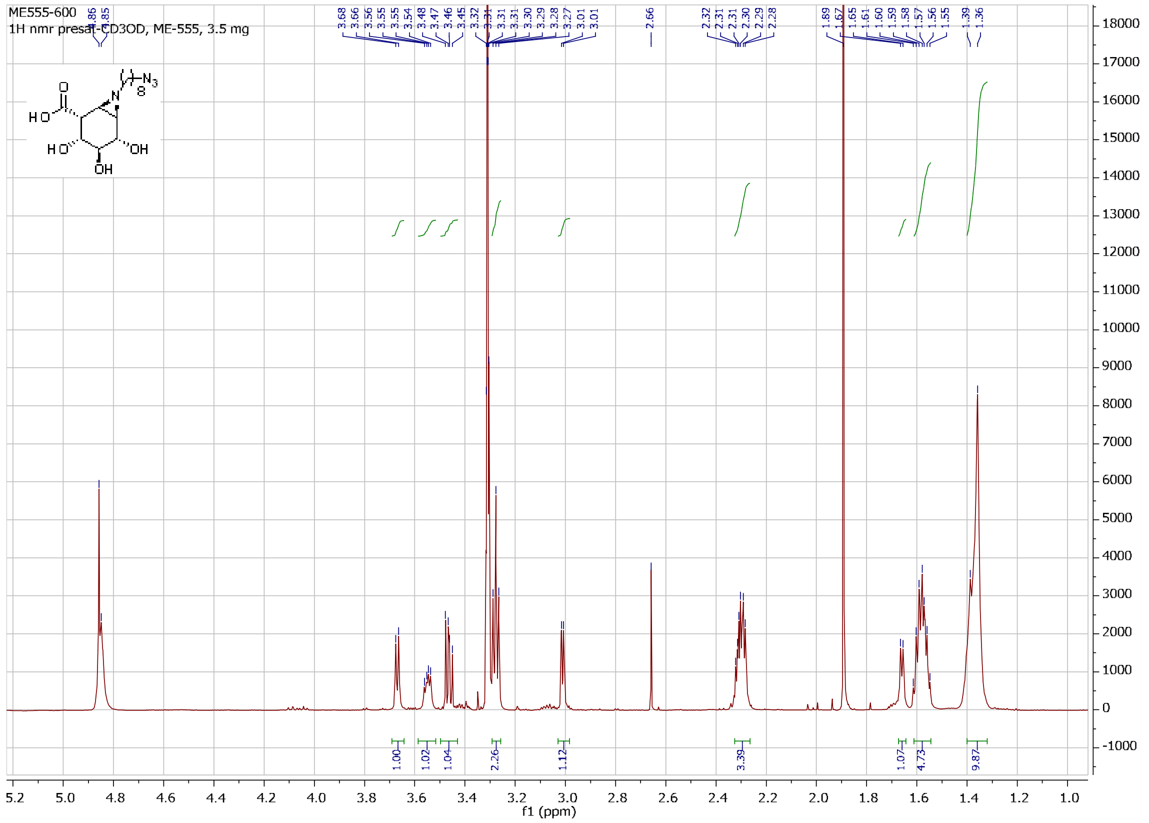

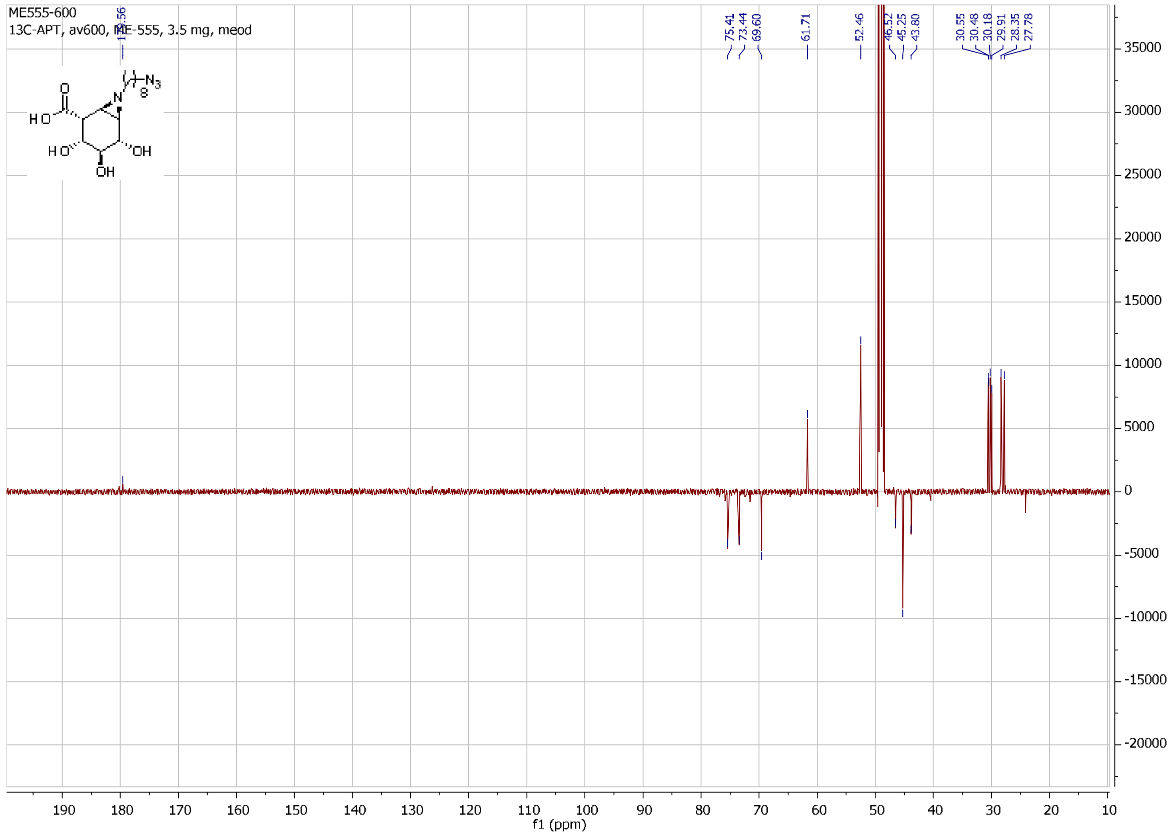

^{1}H-NMR and ^{13}C-NMR spectra of **2** in MeOD

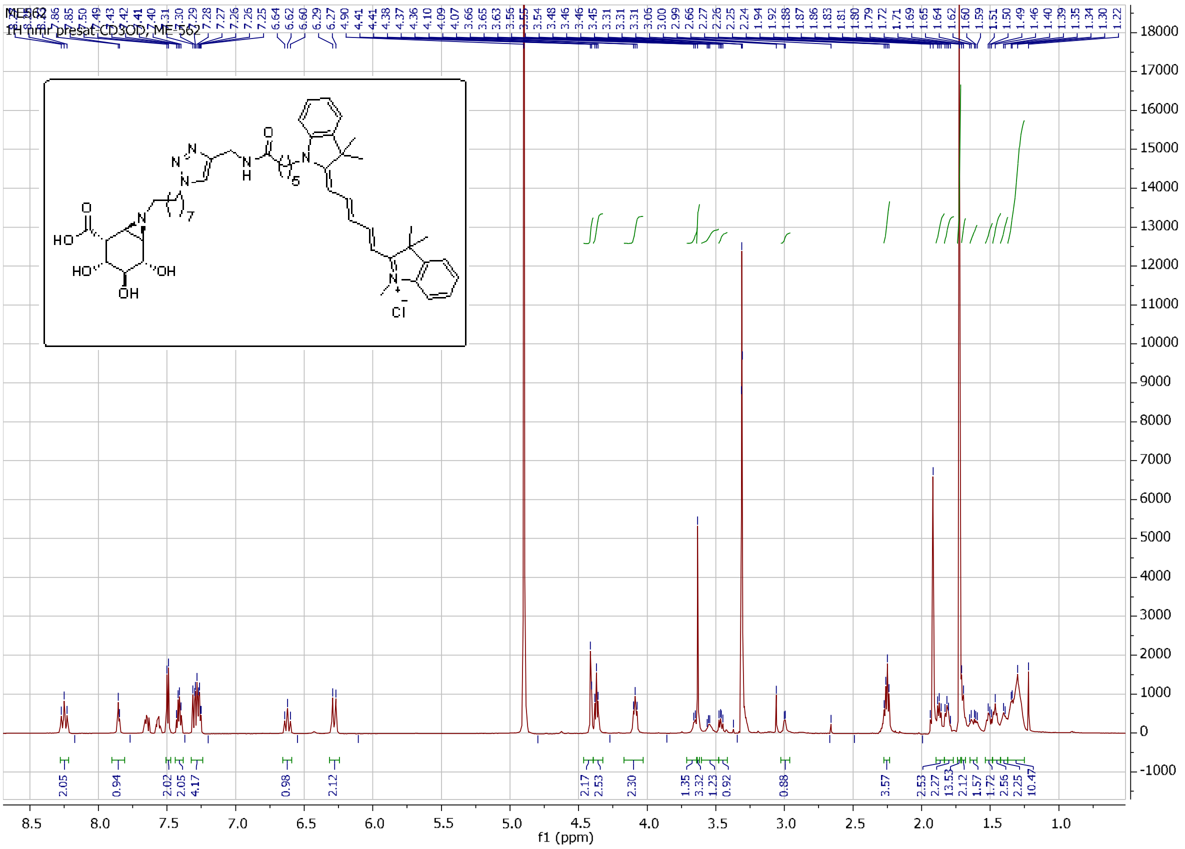

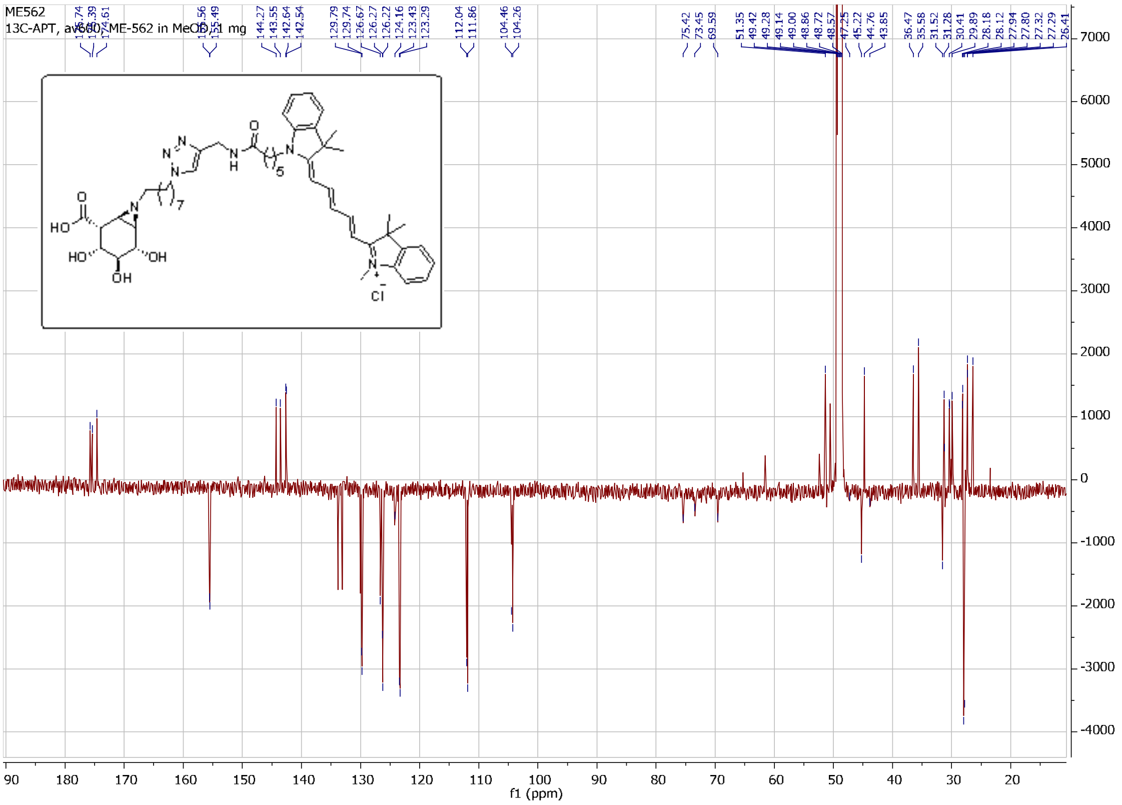

^{1}H-NMR and ^{13}C-NMR spectra of **3** in MeOD

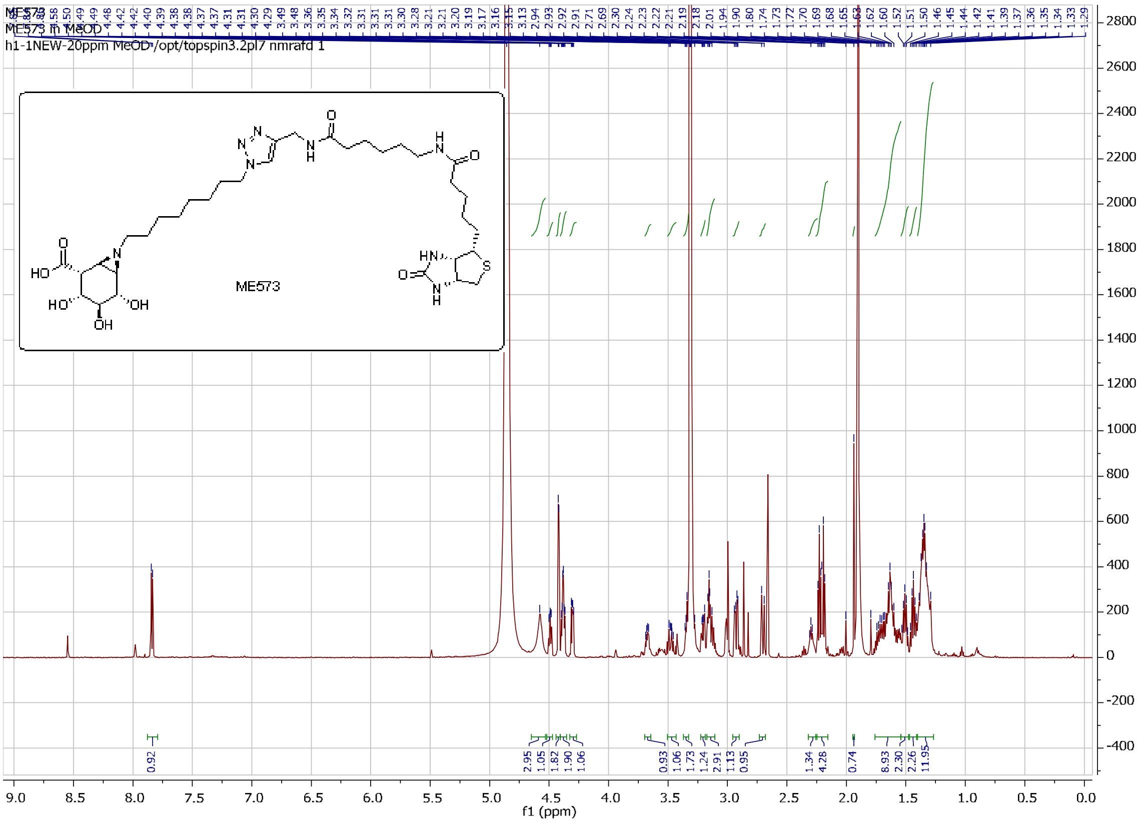

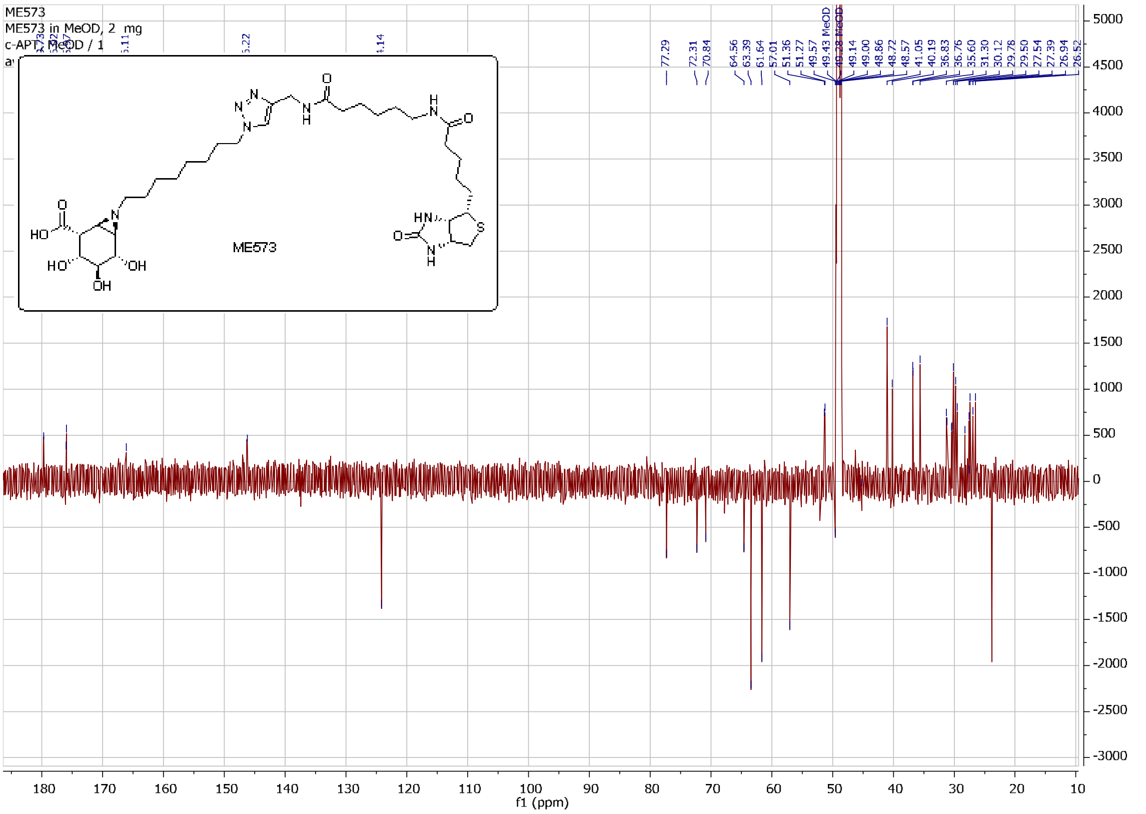

4. References

(1) Ou, L.; Herzog, T. L.; Wilmot, C. M.; Whitley, C. B. Standardization of alpha-L-iduronidase enzyme assay with Michaelis-Menten kinetics. *Mol. Genet. Metab.* 2014, **111** (2), 113-115.

(2) Schröder, S. P.; De Sande, J. W. V.; Kallemeijn, W. W.; Kuo, C. L.; Artola, M.; van Rooden, E. J.; Jiang, J. B.; Beenakker, T. J. M.; Florea, B. I.; Offen, W. A.et al. Towards broad spectrum activity-based glycosidase probes: synthesis and evaluation of deoxygenated cyclophellitol aziridines. *Chem. Commun.* 2017, **53** (93), 12528-12531.

(3) Kuo, C. L.; van Meel, E.; Kytidou, K.; Kallemeijn, W. W.; Witte, M.; Overkleeft, H. S.; Artola, M. E.; Aerts, J. M. Activity-Based Probes for Glycosidases: Profiling and Other Applications. *Methods Enzymol.* 2018, **598**, 217-235.

(4) Li, N.; Kuo, C. L.; Paniagua, G.; van den Elst, H.; Verdoes, M.; Willems, L. I.; van der Linden, W. A.; Ruben, M.; van Genderen, E.; Gubbens, J.et al. Relative quantification of proteasome activity by activity-based protein profiling and LC-MS/MS. *Nat. Protoc.* 2013, **8** (6), 1155-1168.

(5) He, X.; Pierce, O.; Haselhorst, T.; von Itzstein, M.; Kolarich, D.; Packer, N. H.; Gloster, T. M.; Vocadlo, D. J.; Qian, Y.; Brooks, D.et al. Characterization and downstream mannose phosphorylation of human recombinant alpha-L-iduronidase produced in Arabidopsis complex glycan-deficient (cgl) seeds. *Plant Biotechnol. J.* 2013, **11** (9), 1034-1043.

(6) He, X.; Haselhorst, T.; von Itzstein, M.; Kolarich, D.; Packer, N. H.; Gloster, T. M.; Vocadlo, D. J.; Clarke, L. A.; Qian, Y.; Kermode, A. R. Production of alpha-L-iduronidase in maize for the potential treatment of a human lysosomal storage disease. *Nat. Commun.* 2012, **3**, 1062.

(7) He, X.; Haselhorst, T.; von Itzstein, M.; Kolarich, D.; Packer, N. H.; Kermode, A. R. Influence of an ER-retention signal on the N-glycosylation of recombinant human alpha-L-iduronidase generated in seeds of Arabidopsis. *Plant Mol. Biol.* 2012, **79** (1-2), 157-169.

(8) Zhao, K. W.; Faull, K. F.; Kakkis, E. D.; Neufeld, E. F. Carbohydrate structures of recombinant human alpha-L-iduronidase secreted by Chinese hamster ovary cells. *J. Biol. Chem.* 1997, **272** (36), 22758-22765.

(9) Pierce, O. M.; McNair, G. R.; He, X.; Kajiura, H.; Fujiyama, K.; Kermode, A. R. N-glycan structures and downstream mannose-phosphorylation of plant recombinant human alpha-L-iduronidase: toward development of enzyme replacement therapy for mucopolysaccharidosis I. *Plant. Mol. Biol.* 2017, **95** (6), 593-606.

(10) Bie, H.; Yin, J.; He, X.; Kermode, A. R.; Goddard-Borger, E. D.; Withers, S. G.; James, M. N. Insights into mucopolysaccharidosis I from the structure and action of alpha-L-iduronidase. *Nat. Chem. Biol.* 2013, **9** (11), 739-745.

(11) Winter, G.; McAuley, K. E. Automated data collection for macromolecular crystallography. *Methods* 2011, **55** (1), 81-93.

(12) Kabsch, W. XDS. *Acta Crystallogr. D Biol. Crystallogr.* 2010, **66** (Pt 2), 125-132.

(13) Evans, P. R.; Murshudov, G. N. How good are my data and what is the resolution? *Acta Crystallogr. D Biol. Crystallogr.* 2013, **69** (Pt 7), 1204-1214.

(14) Winter, G. xia2: an expert system for macromolecular crystallography data reduction. *J. Appl. Crystallogr.* 2010, **43**, 186-190.

(15) McCoy, A. J. Solving structures of protein complexes by molecular replacement with Phaser. *Acta Crystallogr. D Biol. Crystallogr.* 2007, **63** (Pt 1), 32-41.

(16) Murshudov, G. N.; Skubak, P.; Lebedev, A. A.; Pannu, N. S.; Steiner, R. A.; Nicholls, R. A.; Winn, M. D.; Long, F.; Vagin, A. A. REFMAC5 for the refinement of macromolecular crystal structures. *Acta Crystallogr. D Biol. Crystallogr.* 2011, **67** (Pt 4), 355-367.

(17) Emsley, P.; Cowtan, K. Coot: model-building tools for molecular graphics. *Acta Crystallogr D Biol Crystallogr* 2004, **60** (Pt 12 Pt 1), 2126-2132.

(18) Joosten, R. P.; Long, F.; Murshudov, G. N.; Perrakis, A. The PDB_REDO server for macromolecular structure model optimization. *IUCrJ* 2014, **1** (Pt 4), 213-220.

(19) Bienfait, B.; Ertl, P. JSME: a free molecule editor in JavaScript. *J. Cheminform.* 2013, **5**, 24.

(20) Schuttelkopf, A. W.; van Aalten, D. M. PRODRG: a tool for high-throughput crystallography of protein-ligand complexes. *Acta Crystallogr. D Biol. Crystallogr.* 2004, **60** (Pt 8), 1355-1363.

(21) Spartan ’10, K., J. *et. al.*, Wavefunction Inc., **2004**.

(22) Gaussian 03, M. J. F., G. W. Trucks, H. B. Schlegel, G. E. Scuseria, M. A. Robb, J. R. Cheeseman, J. A. Montgomery, Jr., T. Vreven, *et al.,* Gaussian, Inc., Wallingford CT, **2004**.

(23) C. Y. Legault, C., 1.0b, Univ. Sherbrooke, **2009** (http//www.cylview.org). .

(24) Bally, T.; Rablen, P. R. Quantum-chemical simulation of 1H NMR spectra. 2. Comparison of DFT-based procedures for computing proton-proton coupling constants in organic molecules. *J. Org. Chem.* 2011, **76** (12), 4818-4830.

(25) Hansen, F. G.; Bundgaard, E.; Madsen, R. A short synthesis of (+)-cyclophellitol. *J. Org. Chem.* 2005, **70** (24), 10139-10142.

(26) Evans, D. A.; Sjogren, E. B.; Bartroli, J.; Dow, R. L. Aldol Addition-Reactions of Chiral Crotonate Imides. *Tetrahedron Lett.* 1986, **27** (41), 4957-4960.

(27) Trapero, A.; Llebaria, A. The myo-1,2-Diaminocyclitol Scaffold Defines Potent Glucocerebrosidase Activators and Promising Pharmacological Chaperones for Gaucher Disease. *ACS Med. Chem. Lett.* 2011, **2** (8), 614-619.

(28) Speciale, G.; Thompson, A. J.; Davies, G. J.; Williams, S. J. Dissecting conformational contributions to glycosidase catalysis and inhibition. *Curr. Opin. Struct. Biol.* 2014, **28**, 1-13.

(29) Artola, M.; Wu, L.; Ferraz, M. J.; Kuo, C. L.; Raich, L.; Breen, I. Z.; Offen, W. A.; Codee, J. D. C.; van der Marel, G. A.; Rovira, C.et al. 1,6-Cyclophellitol Cyclosulfates: A New Class of Irreversible Glycosidase Inhibitor. *ACS Cent. Sci.* 2017, **3** (7), 784-793.

(30) Harrak, Y.; Barra, C. M.; Delgado, A.; Castano, A. R.; Llebaria, A. Galacto-configured aminocyclitol phytoceramides are potent in vivo invariant natural killer T cell stimulators. *J. Am. Chem. Soc.* 2011, **133** (31), 12079-12084.

(31) Jaramillo, C.; Delapradilla, R. F.; Martinlomas, M. Synthesis of 1d-1,2-Anhydro-Myo-Inositol. *Carbohyd. Res.* 1991, **209**, 296-298.

(32) Schröder, S. P.; Petracca, R.; Minnee, H.; Artola, M.; Aerts, J. M. F. G.; Codee, J. D. C.; van der Marel, G. A.; Overkleeft, H. S. A Divergent Synthesis of L-arabino- and D-xylo-Configured Cyclophellitol Epoxides and Aziridines. *Eur. J. Org. Chem.* 2016, (28), 4787-4794.

(33) Atkinson, R. S.; Coogan, M. P.; Cornell, C. L. Aziridination of alkenes using 3-acetoxyamino-2-trifluoromethylquinazolin-4(3*H*)-one. *J. Chem. Soc. Perk. T. 1* 1996, 157-166.

(34) Tatsuta, K.; Niwata, Y.; Umezawa, K.; Toshima, K.; Nakata, M. Syntheses and enzyme inhibiting activities of cyclophellitol analogs. *J. Antibiot. (Tokyo)* 1991, **44** (8), 912-914.

(35) Nakata, M.; Chong, C.; Niwata, Y.; Toshima, K.; Tatsuta, K. A family of cyclophellitol analogs: synthesis and evaluation. *J. Antibiot. (Tokyo)* 1993, **46** (12), 1919-1922.

(36) Willems, L. I.; Beenakker, T. J.; Murray, B.; Scheij, S.; Kallemeijn, W. W.; Boot, R. G.; Verhoek, M.; Donker-Koopman, W. E.; Ferraz, M. J.; van Rijssel, E. R.et al. Potent and selective activity-based probes for GH27 human retaining alpha-galactosidases. *J. Am. Chem. Soc.* 2014, **136** (33), 11622-11625.

(37) Caron, G.; Withers, S. G. Conduritol aziridine: a new mechanism-based glucosidase inactivator. *Biochem. Biophys. Res. Commun.* 1989, **163** (1), 495-499.
